# ELAV/Hu RNA binding proteins determine multiple programs of neural alternative splicing

**DOI:** 10.1371/journal.pgen.1009439

**Published:** 2021-04-07

**Authors:** Seungjae Lee, Lu Wei, Binglong Zhang, Raeann Goering, Sonali Majumdar, Jiayu Wen, J. Matthew Taliaferro, Eric C. Lai

**Affiliations:** 1 Developmental Biology Program, Sloan Kettering Institute, New York City, New York, United States of America; 2 Department of Biochemistry and Molecular Genetics, University of Colorado Anschutz Medical Campus, Aurora, Colorado, United States of America; 3 RNA Bioscience Initiative University of Colorado Anschutz Medical Campus, Aurora, Colorado, United States of America; 4 Department of Genome Sciences, The John Curtin School of Medical Research, The Australian National University, Canberra, Australia; University of Basel, SWITZERLAND

## Abstract

ELAV/Hu factors are conserved RNA binding proteins (RBPs) that play diverse roles in mRNA processing and regulation. The founding member, *Drosophila* Elav, was recognized as a vital neural factor 35 years ago. Nevertheless, little was known about its impacts on the transcriptome, and potential functional overlap with its paralogs. Building on our recent findings that neural-specific lengthened 3’ UTR isoforms are co-determined by ELAV/Hu factors, we address their impacts on splicing. While only a few splicing targets of *Drosophila* are known, ectopic expression of each of the three family members (Elav, Fne and Rbp9) alters hundreds of cassette exon and alternative last exon (ALE) splicing choices. Reciprocally, double mutants of *elav/fne*, but not *elav* alone, exhibit opposite effects on both classes of regulated mRNA processing events in larval CNS. While manipulation of *Drosophila* ELAV/Hu RBPs induces both exon skipping and inclusion, characteristic ELAV/Hu motifs are enriched only within introns flanking exons that are suppressed by ELAV/Hu factors. Moreover, the roles of ELAV/Hu factors in global promotion of distal ALE splicing are mechanistically linked to terminal 3’ UTR extensions in neurons, since both processes involve bypass of proximal polyadenylation signals linked to ELAV/Hu motifs downstream of cleavage sites. We corroborate the direct action of Elav in diverse modes of mRNA processing using RRM-dependent Elav-CLIP data from S2 cells. Finally, we provide evidence for conservation in mammalian neurons, which undergo broad programs of distal ALE and APA lengthening, linked to ELAV/Hu motifs downstream of regulated polyadenylation sites. Overall, ELAV/Hu RBPs orchestrate multiple broad programs of neuronal mRNA processing and isoform diversification in *Drosophila* and mammalian neurons.

## Author summary

ELAV/Hu factors comprise a conserved family of RNA binding proteins (RBPs), many of which are enriched or restricted to neurons. This study shows that overlapping activities of *Drosophila* ELAV/Hu RBPs determine global patterns of neural alternative splicing, including of cassette exons and alternative last exon (ALE) isoforms. This is supported by both genetic necessity in double mutant CNS, as well as their sufficiency to drive these mRNA processing changes in a non-neuronal setting. The ability of ELAV/Hu RBPs to induce the usage of distal ALE isoforms connects to their recently described functions in global extension of 3’UTRs in neural isoforms. Evidence is provided that switching to distal alternative last exons and to terminal 3’ UTR extensions are also coordinated in mammalian neurons, and show signatures of direct regulation by ELAV/Hu RBPs.

## Introduction

**Fig 1:**
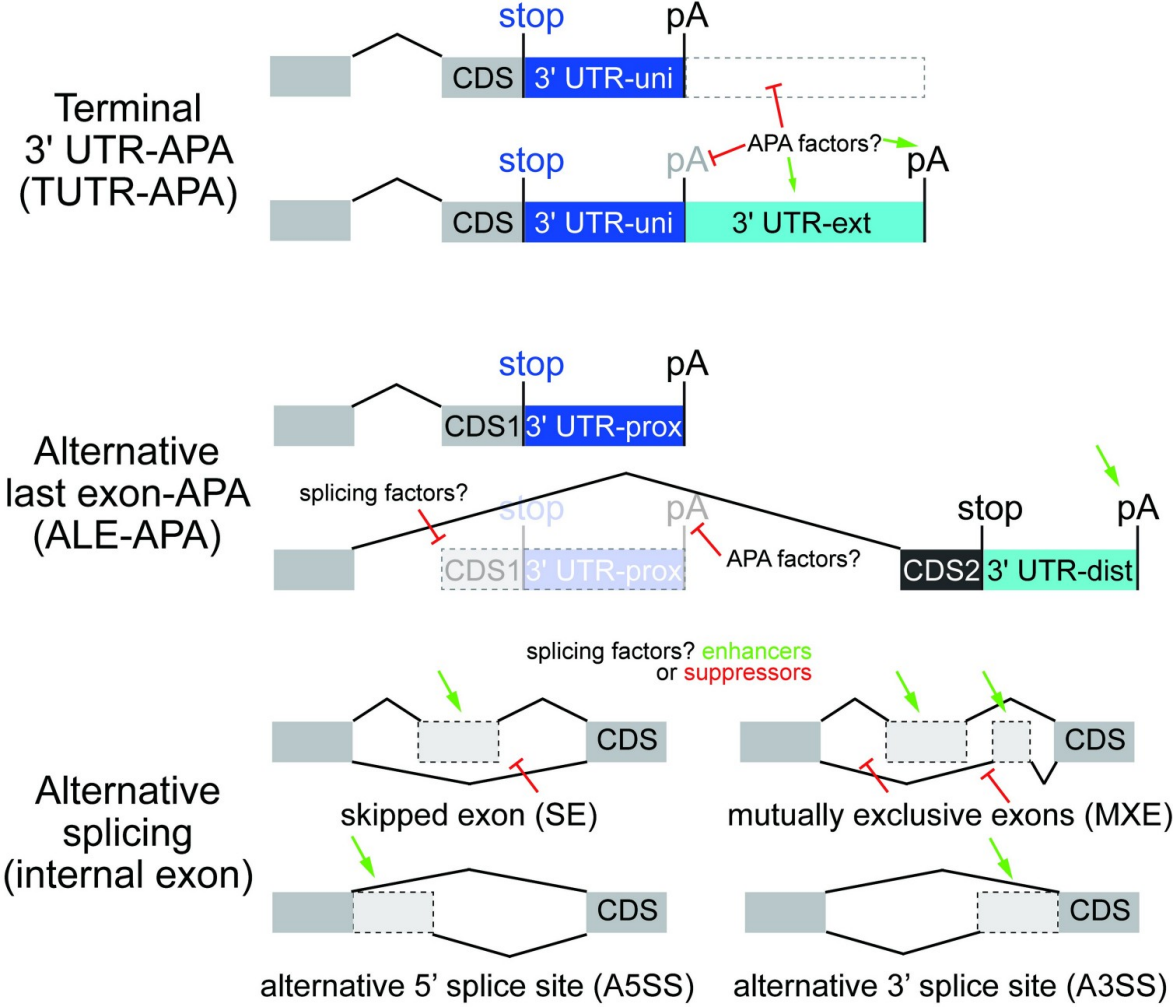
Summary of alternative polyadenylation and alternative splicing. Top, Alternative polyadenylation (APA) within the terminal 3’ UTR (TUTR-APA) generates 3’ UTR isoforms of variable lengths (universal/uni vs. extension/ext). Amongst other mechanisms, APA factors might modulate recognition of proximal pA signals, or selectively modulate the stability of ext isoforms. Middle, Alternative last exon (ALE)-APA generates isoforms with variable coding last exons, along with entirely distinct 3’ UTRs (proximal/prox vs. distal/dist). In principle, these could be regulated by splicing recognition, or by pA site recognition. Bottom, Alternative splicing of internal exons can be classified into several groups, and can be influenced by splicing enhancer or suppressors that bind in exonic or intronic regions. Not shown, alternative 5’ exons generated by distinct transcriptional start sites. This study will focus on exon skipping and the mechanistic relationship of TUTR-APA and ALE-APA.

The vast majority of genes in higher eukaryotes are subject to a variety of alternative processing mechanisms that diversify the functional outputs of the transcriptome [1,2]. The usage of alternative promoters, engagement of distinct internal and/or last exons by alternative splicing, and the utilization of alternative polyadenylation signals (PAS), can collectively generate transcript isoforms that differ in 5’ UTRs, coding exons, and/or 3’ UTRs (**Fig 1**). These regulatory regimes have broad consequences for differential regulation of isoforms as well as to broaden the protein outputs of an individual locus, and are aberrant in disease and cancer [3,4].

While all of these regulatory concepts are applicable to all tissues and cells, the nervous system is well-known for exuberant deployment of alternative isoforms [5,6]. For example, vertebrate brains encode the most diverse and conserved sets of alternative splicing events [7,8]. An extreme example is alternative splicing of the *Drosophila* immunoglobulin superfamily gene *Dscam*, which can generate ~38,000 distinct proteins [9]. Indeed, experimental manipulations demonstrate that a high diversity of isoforms is functionally required for neuronal wiring [10]. The nervous system also exhibits broad usage of other unusual splicing programs. For example, mammalian brains preferentially express a suite of short "microexons" (encoding <10aa), which appear to be dysregulated in neurological disease [11]. Back-splicing that generates circular RNAs can be considered as another type of alternative splicing, and this class of species is most abundant and diverse in the nervous system [12,13]. Finally, *Drosophila* and vertebrate CNS exhibit the longest 3’ UTRs of any tissue [14–16].

Splicing and polyadenylation are catalyzed by large multisubunit RNA processing complexes, the former by the spliceosome and the latter by the cleavage and polyadenylation (CPA) machinery. As may be expected, direct experimental manipulations of core spliceosome or polyadenylation factors have broad impacts to deregulate isoform choice. The levels of many core splicing and polyadenylation factors differ across development and/or tissue or cell type, suggesting that their endogenous modulation may in part be linked to alternative mRNA processing. Reciprocally, trans-acting factors can impinge on the activities of splicing or polyadenylation machineries, either positively or negatively, to confer alternative processing.

The diversity of molecular processing mechanisms is best understood for alternative splicing [1,17]. Most of these strategies influence the definition of exons or introns, resulting in differential inclusion or exclusion of sequences in mature mRNAs (**Fig 1**). The SR family of RNA binding proteins (RBPs) provide a paradigm for proteins that bind exonic sequences to promote their inclusion, but certain SR factors bind intronic sequences to promote exon exclusion [18]. Many other non-SR proteins can regulate splicing (e.g. Nova, ELAV/Hu factors, PTBP proteins, MBNL proteins, etc.), and they generally mediate their effects by binding in the vicinity of splice sites. Notably, several of these have position-specific effects. For example, RBP binding overlapping a splice site can block exon inclusion, but intronic binding of the same factor can also promote exon inclusion [19–22].

Regarding APA, there are numerous ways that core CPA and trans-acting factors that influence sites of 3’ cleavage, and these can have distinct impacts depending on location within the gene [2,4]. When multiple pA sites are differentially utilized in the same terminal 3’ UTR exon ("TUTR-APA", also referred to as tandem APA), this results in inclusion or exclusion of cis-regulatory sites within nested 3’ UTRs (**Fig 1**). The length of alternative 3’ UTRs can be quite substantial (>10 kb), and there may be sequence-independent effects of 3’ UTR length variants. Another general class of APA events occur within regions that are internal to the last coding exon of the longest gene model. These might occur within internal exons or introns, and have variously been termed upstream region-APA, intronic APA, coding sequence-APA (CDS-APA) or alternative last exon (ALE)-APA; we will use the latter designation in this study.

If internal polyadenylation impairs the stability of the alternative transcript, this can result in loss-of-function of that isoform. However, ALE-APA can also produce alternative stable transcripts, which encode distinct protein isoforms (**Fig 1**). In some cases, internally polyadenylated isoforms yield proteins that lack C-terminal domains of "full-length" counterparts, which might inactivate them and/or encode dominant negative or neomorphic activities [23]. On the other hand, there are many examples of loci with distinct ALE isoforms that harbor different activities, analogous to distinct internal splice isoforms. Indeed, early characterizations of alternative polyadenylation involved ALE isoforms that switch the localization of IgM heavy chain products from secreted to membrane-bound [24], or generate distinct protein isoforms of calcitonin/CGRP[25]. Beyond distinct coding potential, different ALE isoforms can also be subject to regulated subcellular localization, particularly within neurons [26–28]. Although less is understood about trans-acting factors that influence APA, several RBPs (e.g. PABPN1, CELF1, etc.) operate in a conceptually similar manner to splicing regulators. Namely, several RBPs modulate 3’-end selection by binding in the vicinity of cleavage sites to oppose the action of the CPA machinery [29–33].

The ELAV/Hu family of RBPs are conserved across metazoans and play broad roles in RNA biogenesis, including alternative splicing, APA, target stability, translation, and localization [34–41]. In *Drosophila*, Elav was proposed as a direct regulator of both splicing and APA. Notably, there is a conceptual mechanistic link between these regulatory processes. The first direct target of Elav characterized was *erect wing* (*ewg*), and it controls splicing of its alternative last exons [40]. Elav also regulates the splicing of certain internal exons [42,43], but unlike other strategies for splicing control mentioned above, Elav controls neural-specific *ewg* by modulating cleavage and polyadenylation. In particular, Elav suppresses cleavage at the 3’ end of an internal *ewg* terminal exon isoform, thereby permitting transcription to extend to the distal terminal exon. This mechanism also applies to neural-specific of *neuroglian* (*nrg*) [41]. An analogous function for Elav was reported to promote neural-specific 3’ UTR lengthening at select genes bearing tandem polyA signals within the same 3’ UTR [38]. In this setting, it was proposed that association of Elav with a proximal polyA signal inhibits processing by the CPA machinery, thereby permitting transcription into distal 3’ UTR segments.

These studies focused on regulation of a few specific *Drosophila* genes, but broader impacts of Elav and/or its paralogs on the mRNA processing were not yet addressed. In work published during review of this manuscript, we made the following observations [44]. First, although Elav has long been considered to be embryonic lethal, we found that *elav* deletion mutants are nominally viable as 1st instar larvae. With access to *elav* null larval CNS we found that its complete loss was compatible with expression of many neural APA 3’ UTR extensions. Second, we found using gain-of-function strategies, that all three *Drosophila* ELAV/Hu members (Elav, Fne, Rbp9) have similar capacities to induce a neural 3’ UTR extension landscape in an ectopic setting (S2 cells). Third, we found that functional redundancy is endogenously relevant, because elav/fne double mutant larval CNS exhibit a severe loss of neural 3’ UTR extension landscape. Fourth, the functional overlap of Elav and Fne involves a regulatory interplay, because Elav represses *fne* alternative splicing that switches it from a cytoplasmic to a nuclear isoform. The hierarchical role of Elav and Fne in neural mRNA processing was also reported in a contemporary study by the Hilgers group [45].

Here, we exploit gain-of-function and loss-of-function genomic datasets to study the impact of *Drosophila* ELAV/Hu factors on alternative splicing, including both internal exons as well as terminal exons. We broaden the set of cassette exons and alternative 5’ or 3’ splice sites that are regulated by Elav and Fne from just a few to many hundreds. Moreover, we show that overlapping activities of ELAV/Hu factors are necessary and sufficient to define a broad program of neural ALE splicing. Genomic analyses reveal mechanistic parallels between neural ALE splicing and neural 3’ UTR lengthening, demonstrating that these are analogous processes that operate in a directional manner on transcripts to promote the inclusion of distal exonic sequences in neurons. Finally, we extend these findings to mammals, and provide evidence for coincident shifts towards usage of distal ALE isoforms and extended tandem 3’ UTRs during directed neuronal differentiation, coupled to enrichment of ELAV/Hu motifs at bypassed pA sites. This indicates conservation and coordination of these two RNA processing pathways across metazoan neurons.

## Results

### All *Drosophila* ELAV/Hu factors can induce broad programs of alternative splicing

Mammalian ELAV/Hu factors are documented regulators of alternative splicing, mediating both exon inclusion and exclusion. This applies to both ubiquitously expressed HuR [46,47] as well as neuronally restricted HuB/C/D [19,48]. However, even though *Drosophila* Elav was shown to be involved in splicing [49] before its mammalian counterparts [50], until recently, the internal splicing of only two *Drosophila* genes (*Dscam1* and *arm*) was known to be dependent on Elav [42,43]. Thus, it was unclear if splicing is a diverged regulatory function for ELAV/Hu RBPs. Moreover, the capacity of other *Drosophila* ELAV/Hu paralogs (Fne and Rbp9) to influence splicing was largely unknown, but presumed not endogenously relevant due to their largely cytoplasmic localization [51,52].

**Fig 2:**
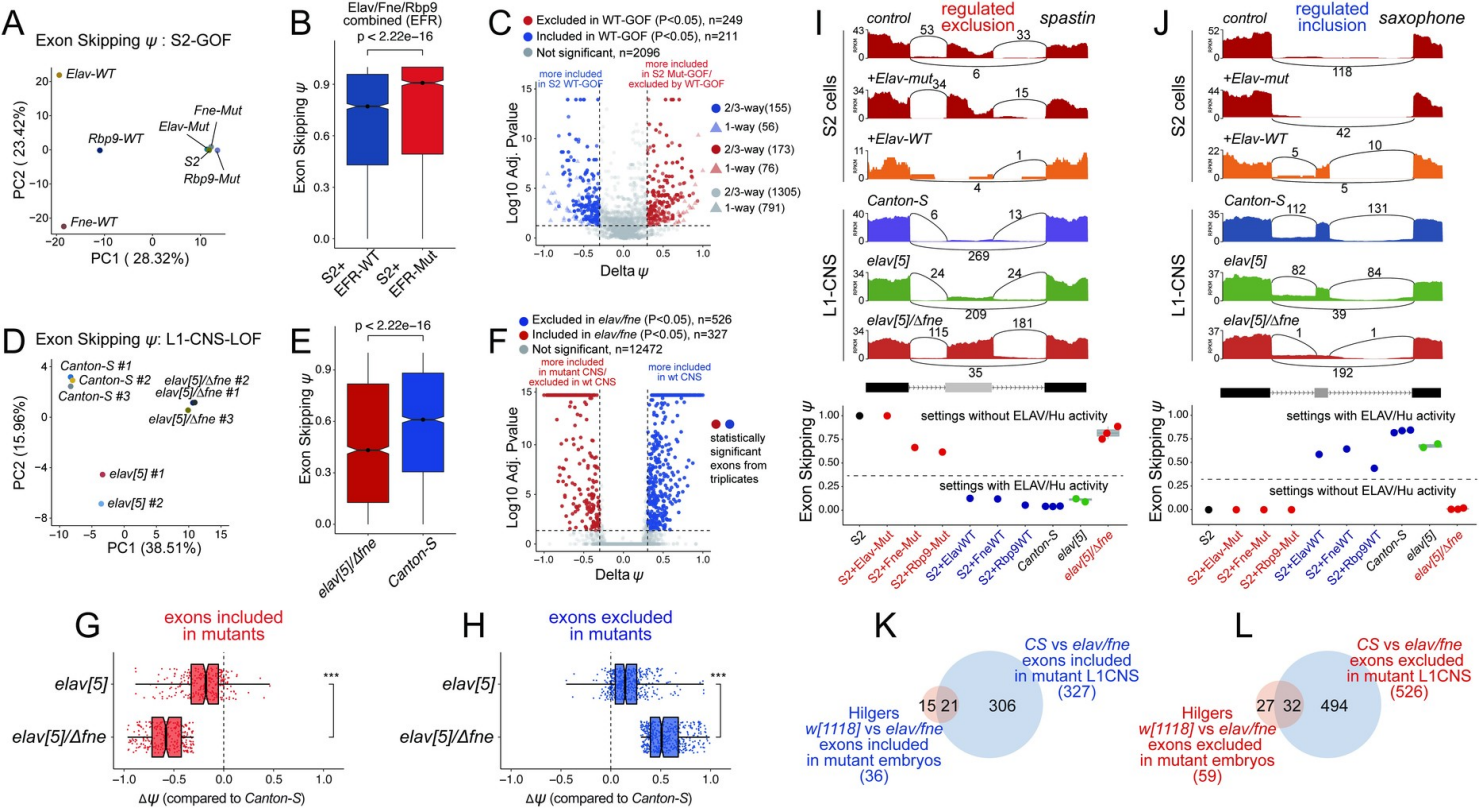
Overlapping activities of *Drosophila* ELAV/Hu RBPs control alternative cassette exon splicing. (A) Principal components analysis (PCA) of alternative splicing of cassette exons (percent spliced in, PSI/*ψ* values) RNA-seq data from S2 cells transfected with wildtype (wt) or 3xRRM-mut (mut) versions of Elav, Fne and Rbp9 (i.e., EFR). (B) Fraction of exon skipping events in averaged S2 EFR-wt or EFR-mut datasets, treating the different ELAV/Hu RBP conditions as pseudo-replicates. (C) Volcano plot of alternative spliced cassette exon events in S2 cells following transfection of ELAV/Hu RBPs. Note that exons preferentially excluded by wt-ELAV/Hu RBPs correspond to exons that are preferentially included in control S2 cells and cells expressing mutant ELAV/Hu RBPs, and *vice versa*. rMATs will calculate P-values without replicates [53], but the values determined from replicate data are more confident. Thus, we distinguish exons with similar responses between 3, or across all three ELAV/Hu datasets. (D) PCA of cassette exon splicing in L1-CNS RNA-seq data from *Canton-S*, *elav* mutant, and double *elav/fne* mutants. (E) Fraction of exon skipping events in *elav/fne* mutant and *Canton-S* L1-CNS. (F) Volcano plot of alternatively spliced cassette exon events in *Canton-S* and *elav/fne* mutant L1-CNS. For effective visualization of volcano plots, a pseudocount of 1E-14 was added to all datapoints, to allow plotting of loci with P-value of 0; the actual values are shown in S1 Table. (G-H) The magnitude of splicing changes, for both included (G) and excluded (H) exons, is far greater in *elav/fne* double mutants than *elav* single mutants. *ψ* values in both mutants were calculated relative to *Canton-S*. (I) Example of a novel exon exclusion target of ELAV/Hu RBPs. (J) Example of a novel exon inclusion target of ELAV/Hu RBPs. (K-L) Comparison of statistically significant cassette exons in dissected L1-CNS, with those annotated from whole embryos [45].

We investigated if these RBPs have broader impacts on alternative splicing. We first generated RNA-seq datasets from S2 cells that ectopically expressed wt Elav/Fne/Rbp9, or RNA binding-defective variants bearing inactivating point mutations in all three RRM domains (referred to as "mut"). We then used rMATS [53] to investigate various classes of alternative splicing; this package classifies alternative internal isoforms (**Fig 1**). This revealed 610 differentially spliced exons across all alternative splice types, and relatively comparable numbers of exons exhibited gains or losses of usage in the presence of ectopic ELAV/Hu factors (**S1 Fig**). Exon skipping comprised the major deregulated category, and these exhibited mild directional bias for exclusion by ectopic wt ELAV/Hu factors compared to their mutant variants, but substantial numbers of exons were driven towards inclusion (**S1 Fig**). Since cassette exons were the dominant source of alternative splicing, we performed principal components analysis (PCA) on exon skipping. This showed that mutant ELAV/Hu datasets clustered near control S2 cells, while wildtype Elav/Fne/Rbp9 were well-separated (**Fig 2A**). Therefore, ectopic ELAV/Hu proteins induced substantial alternative splicing programs that were dependent on their RNA binding activities. We observed substantial overlap in the regulatory influences of these factors (**S1 Fig**, with examples of individual genes in **S2 Fig**). As we had single RNA-seq datasets for each RBP, we focused on the extensive set of cassette exons with similar differential splicing responses, which comprise robust targets of overlapping capacities of the ELAV/Hu RBP family (**Fig 2B and 2C**).

### Combined endogenous activities of Elav and Fne determine neural alternative splicing

Since the S2 cell system is an ectopic setting, we addressed the endogenous impact of ELAV/Hu RBPs on splicing using recently available RNA-seq data from first larval instar (L1)-CNS [44]. We found that L1-CNS is a setting in which there is substantial functional overlap of Elav and Fne to direct neural 3’ UTR extensions, especially as a consequence of nuclear re-localization of Fne in *elav* mutants [44]. Interestingly, this involves a regulatory interplay of these factors, since Elav represses a novel alternative splice isoform of *fne* that is nuclearly localized. As Fne is neural-specific, this regulatory paradigm could not be appreciated from S2 cells. Since we were particularly interested in identifying endogenous splicing targets confidently, which may include other neural-restricted loci, we utilized replicate datasets for these *in vivo* samples. Accordingly, we examined global alternative splicing in L1-CNS from control *Canton-S* (wildtype), *elav[5]* null, and *elav[5]/fne[Δ]* double deletion backgrounds.

As with the S2 analyses, categorization of all alternative splicing events showed that exon skipping compromised the major class (**S1 Fig**). In addition, while *elav* deletion clearly had impact on neural splicing, all effects were more profound in *elav/fne* double mutants. This could be visualized by the greater numbers of affected loci when comparing either *elav* or *elav/fne* to control, or by comparing the single and double mutants (**S1 Fig**). Of note, the breadth of splicing deregulation in the nervous system was even greater than in the ectopic studies. We detected 1624 splicing alterations in double mutants, with cassette exons again representing the majority of events (**S1 Fig**). PCA of alternative cassette exon usage across L1-CNS replicates (**Fig 2D**) highlights the more severe effects of dual deletion of *elav/fne* compared to *elav*. We summarize the impacts of *elav/fne* double mutants on exon skipping in **Fig 2E and 2F**, and highlight that broad splicing deregulation in L1-CNS requires combined loss of ELAV/Hu RBPs, with respect to both exon inclusion and exclusion (**Fig 2G and 2H**). Overall, the combined activity of these ELAV/Hu factors specifies endogenous neural splicing, and we focus on exons deregulated in double mutants loci for further analyses.

We highlight examples from broad and reciprocal splicing dysregulation following manipulation of ELAV/Hu factors in S2 gain-of-function (GOF) and L1-CNS loss-of function (LOF) settings. Loci with exon-skipping promoted by ELAV/Hu factors include the cell adhesion/Wg pathway coactivator *armadillo*, the Dpp pathway transcription factor *Medea* and the microtubule severing factor *spastin*. These loci harbor exons that are included in control S2 cells or cells expressing 3xRRM-mutant versions of Elav/Fne/Rbp9, but are efficiently skipped in cells expressing wildtype Elav/Fne/Rbp9 (**Figs 2I** and **S2**). On the other hand, their exons are skipped in wt and *elav[5]* L1-CNS, but are included in *elav[5]/fne[Δ]* L1-CNS (**Fig 2I**). On the other hand, the BMP receptor *saxophone* (**Fig 2J**), and two genes involved in cytoskeleton remodeling, *LIM kinase 1* and the cysteine peptidase inhibitor *sigmar* (**S2 Fig**), exemplify loci whose cassette exon inclusion is promoted by ELAV/Hu factors, and have opposite behavior in GOF and LOF settings.

While this work was under consideration, Hilgers and colleagues reported that *elav* mutant embryos exhibited splicing defects that were exacerbated by concurrent deletion of *fne* [45]. While these conclusions appear in line with ours, their annotations used RNA-seq data from whole embryos, which are confounded by expression of non-neuronal isoforms layered on top of potentially altered mRNA processing in ELAV/Hu-mutant nervous system. In addition, we noted that Fne protein accumulates to a higher level in L1-CNS relative to embryos [44], so that compensatory roles of ELAV/Hu RBPs might be more overt in more mature neurons from the first larval instar stage. For these reasons, we suspected that whole embryo RNA-seq data would be underpowered to detect the breadth of alternative splicing changes in ELAV/Hu mutants. Indeed, by visual inspection, the magnitude of changes in alternatively spliced exons are frequently much larger in L1-CNS than in whole embryos, and many that were splicing annotated only in embryos fell below the cutoffs we used in our study and/or had marginal changes (**S3 Fig**). Overall, although we used more stringent criteria in our efforts, we detect nearly an order of magnitude more ELAV/Hu-dependent alternatively spliced exons in our mutant L1-CNS datasets than were reported from embryos (**Fig 2K and 2L** and **S3**). The complete cassette exon splicing analyses are provided in **S1 Table**.

Taken together, these analyses reveal that beyond a couple of loci studied over the past 20 years [42,43], *Drosophila* ELAV/Hu factors are global regulators of alternative neural splicing.

### Validation of exon exclusion and inclusion events driven by *Drosophila* ELAV/Hu RBPs

**Fig 3:**
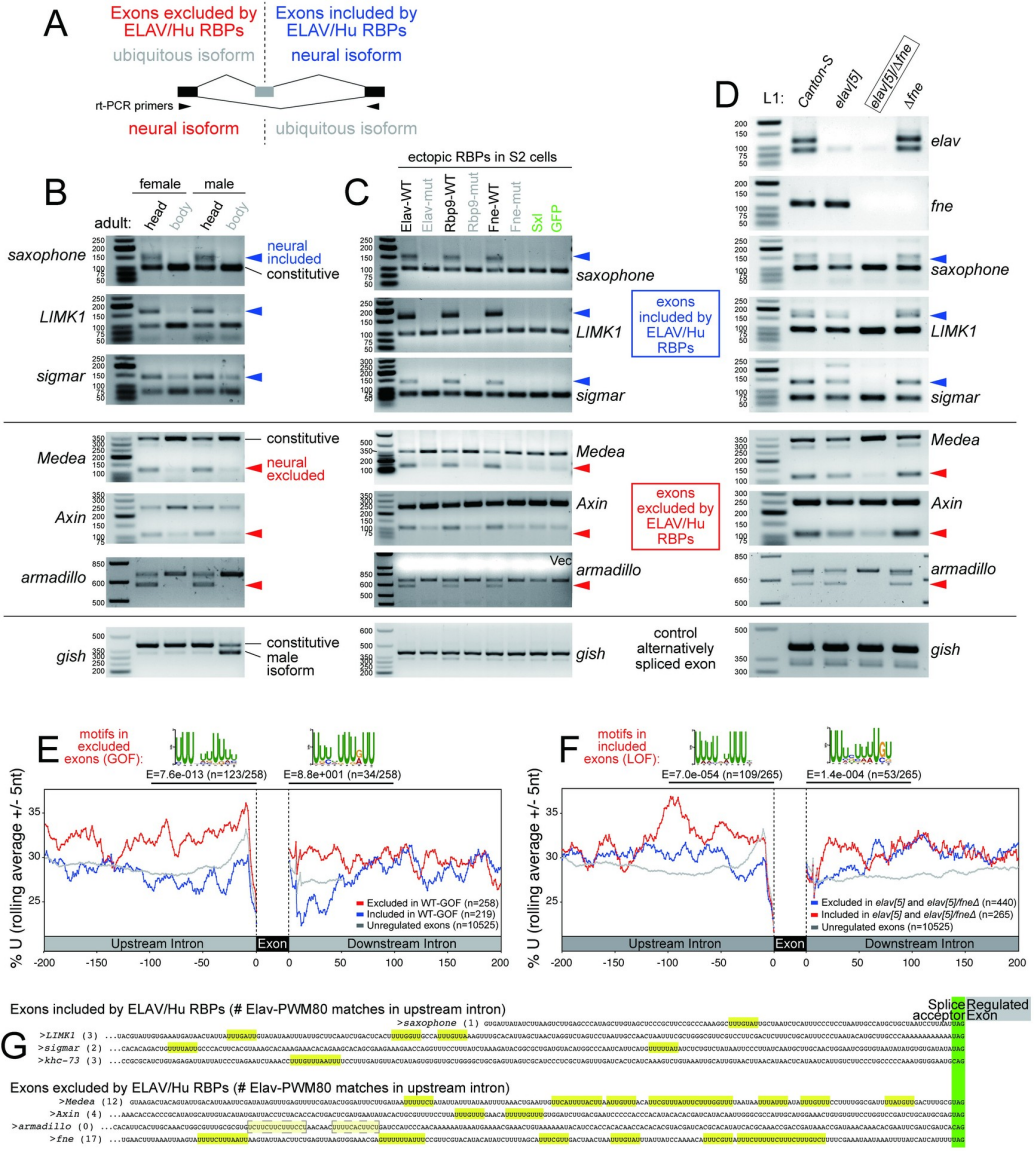
Experimental validation of neural-specific cassette exon splicing regulated by ELAV/Hu family RNA binding proteins. (A) Experimental design and rt-wPCR primers to test neural specific cassette exon splicing. All rt-PCR tests were conducted at least twice with independent RNA samples with similar results; the data shown here are representative. B) rt-PCR validation of cassette exon inclusion (*saxophone*, *LIMK1*, *sigmar*) and exclusion (*Axin*, *Medea*, *armadillo*), no change (*gish*) gene candidates in adult fly tissue samples. (C) rt-PCR validation of cassette exon inclusion (*saxophone*, *LIMK1*, *sigmar*) and exclusion (*Axin*, *Medea*, *armadillo*), no change (*gish*) gene candidates in S2R+ cells transfected with wildtype and RRM-mutant ELAV/Hu RBPs, together with another U-rich binding protein Sxl. (D) rt-PCR validation of cassette exon inclusion (*saxophone*, *LIMK1*, *sigmar*) and exclusion (*Axin*, *Medea*, *armadillo*), no change (*gish*) gene candidates in *Canton-S* and single or double mutant 1^st^ instar larvae (L1). (E) Global analysis of exons excluded by mis-expression of ELAV/Hu RBPs shows U-enrichment in flanking intronic regions, especially in the upstream regions. Comparable U-enrichment is not seen in the vicinity of exons that are included by ELAV/Hu factors. *De novo* motif analysis shows the top enrichment sequence matches the ELAV/Hu binding site. (F) Global analysis of exons included in *elav* and *elav/fne* mutant L1-CNS, compared to *Canton-S*, shows similar positional enrichment of intronic uridine and Elav binding sites. (G) Elav binding motifs found in upstream intron region of exon inclusion and exclusion gene candidates regulated by ELAV/Hu family RBPs (highlighted in yellow, at least 80% matches to the Elav position weight matrix [Elav-PWM80]). There is typically much greater density of ELAV/Hu motifs upstream of excluded exons, compared to included exons.

We performed rt-PCR assays to validate consequences of manipulating ELAV/Hu RBPs on alternative splicing targets, including exons that are excluded or are included in the presence of ELAV/Hu factors, under gain- or loss-of function conditions (**Fig 3A**). To visualize neural-specific alternative splicing, we tested a panel of candidate amplicons in wildtype heads and bodies. We could detect both exon inclusion, revealing longer products for *saxophone*, *LIMK1* and *Sigmar* in heads, as well as exon exclusion, showing enrichment of shorter products for *Medea*, *Axin*, and *armadillo* in heads compared to bodies (**Fig 3B**). As a control, we used *gish*, which has a shorter sex-specific splice isoform that is detected from male bodies, but no differences were seen amongst female heads or bodies, or male heads (**Fig 3B**).

To test sufficiency of regulation by ELAV/Hu factors, we used S2 cells transfected with actin-promoter expression constructs for wildtype or 3xRRM-mutant Elav, Fne and Rbp9. We previously showed these induce overlapping regulatory effects on primary mRNA processing [44]. Thus, even though Elav is normally the dominant nuclear ELAV/Hu member in *Drosophila*, its paralogs can mediate similar nuclear regulatory effects. This caused us to assess the subcellular distribution of ectopic ELAV/Hu proteins. We are aware that immunostaining can be misleading; for example, we found that seemingly neuronal-specific, neural Elav also has lower, ubiquitous, cytoplasmic accumulation, which is overlooked with typical imaging [54]. Instead, we performed cell fractionation to assess this (**S4A Fig**). Ectopic wt and RNA-binding-defective Elav were predominantly nuclear (**S4B Fig**), suggesting that nuclear localization of Elav is not gated by target association. Meanwhile, both Fne and Rbp9 were detected in both cytoplasm and nucleus (**S4B Fig**). Although this is ectopic expression, wildtype heads exhibit substantial nuclear accumulation of Fne protein [55] even though the *fne* exon-included isoform is barely detected. This suggests that *fne* splicing may not be the sole determinant for subcellular control of Fne, and that both Fne isoforms may have capacities for mRNA processing.

With this mind, we evaluated a panel of alternatively spliced targets in S2 cells. We observed that ectopic Elav/Fne/Rbp9 all induced inclusion of cassette exons at *saxophone*, *LIMK1* and *Sigmar*, but had opposite effects on other targets, resulting in exclusion of cassette exons of *Medea*, *Axin* and *armadillo* (**Fig 3C**). Of note, *armadillo* (*arm*) is one of two known cassette exon targets of Elav (exclusion) [42], but the others were not tested previously. We evaluated the specificity of these results using these negative controls. First, we tested transfection controls. We previously showed that other RBPs with U-rich preference cannot induce 3’ UTR extension, as can ELAV/Hu RBPs [44]. Here, we observed that Sex-lethal (Sxl), Ssx, hnRNP-C and CstF64 were not able to alter *LIMK1* splicing, and neither were GFP or empty vector conditions (**S5 Fig**). Of these, we consider Sxl as a stringent test, since it is the closest relative of ELAV/Hu members in *Drosophila* and its overexpression can regulate splicing of an Elav target reporter [55] and we validated its capacity to alter Sxl splicing in S2 cells [56]. Systematic tests showed that neither GFP nor Sxl had any influence on alternative splicing of either inclusion or exclusion targets of ELAV/Hu RBPs (**Fig 3C**). Second, we found that none of these RBPs affected alternative splicing of the control locus *gish* (**Fig 3C**). Thus, we can confirm specific and robust modulation of target exon exclusion and inclusion downstream of all three *Drosophila* ELAV/Hu factors, dependent on their RRMs.

To test necessity of regulation, we analyzed mutant L1 larvae. Of our experimentally validated loci, only *arm* had been previously examined for endogenous regulation, whereby loss of *elav* compromises accumulation of neural-specific *arm* isoform in eye discs [42]. However, in L1 larvae, we saw no substantial changes in the distribution of *arm* isoforms in either *elav* null or *fne* null larval samples (**Fig 3D**). Strikingly, however, rt-PCR from *elav/fne* double mutant larvae showed complete absence of the neural *arm* isoform (**Fig 3D**). Therefore, both Elav and Fne are needed to generate the exon-excluded isoform of *arm* in larval CNS [57]. We extended this to our panel of S2-validated exon inclusion and exclusion targets, and find broad evidence that combined activities of these ELAV/Hu factors are needed to generate neural isoforms. That is, the single null mutants of either RBP had relatively little effects, while the double mutants exhibited complete absence of the included isoforms of *saxophone*, *LIMK1* and *Sigmar*, and showed strong loss of excluded isoforms of *Medea* and *Axin* (**Fig 3D**). Splicing of the negative control *gish* was not affected in any of the mutant genotypes tested (**Fig 3D**).

Overall, these data broadly validate that multiple ELAV/Hu RBPs are both necessary and sufficient to drive neural alternative splicing in *Drosophila*.

### Exons excluded by *Drosophila* ELAV/Hu factors exhibit signatures of direct regulation

Prior analyses in mammals detected numerous alternative splicing events regulated by HuR [46,47] or neuronal Hu proteins [19], but were on the whole equivocal as to the layout of ELAV/Hu-regulated exons. Based on analyses of a few dozen alternative splicing events, it was concluded that binding of ELAV/Hu RBPs is enriched upstream of both included and excluded exons [19,47]. However, it was also suggested that many alternative splicing events detected after HuR depletion were likely indirect effects [47].

We addressed this in our data by analyzing sequence properties and performing *de novo* motif searches amongst different cohorts of alternatively spliced cassette exons. We observed overtly distinct nucleotide distributions depending on whether they were excluded or included, and these exhibited reciprocal features in GOF and LOF data. In particular, exons that were preferentially excluded in S2-ELAV/Hu-GOF data exhibited distinctly elevated U-content in flanking upstream intronic regions, and this was also seen with exons that were preferentially included in *elav/fne* mutant L1-CNS (**Figs 3E and 3F** and **S6**). Importantly, while these regions are expected to have biased content because they overlap polypyrimidine (C/U) tracts located upstream of strong splice acceptor sequences, comparable elevation of U-content was not observed upstream of exons that displayed opposite regulatory behavior in S2 or L1-CNS. Instead, we observed elevated A-content downstream of exons that were preferentially included in S2-ELAV/Hu-GOF settings, or were excluded in *elav/fne* mutant L1-CNS (**S6 Fig**). Therefore, elevated U-content in flanking introns is a specific feature of exons that are excluded by ELAV/Hu factors.

We performed motif discovery on these cohorts of introns, noting that the conserved binding site of ELAV/Hu factors is known to be U-rich [58]. Strikingly, close matches to the empirically-determined binding sites for Elav/Fne/Rbp9 [58] are highly enriched upstream of exons that were ectopically excluded in S2-GOF data, and reciprocally in exons that were ectopically included in *elav/fne* mutant L1-CNS data (**Fig 3E and 3F**). These were the most frequent (~40–50% of regulated exons) and most significant motifs found in the intronic regions upstream of exons regulated in these directions; they were also significantly enriched downstream of these regulated exons, albeit less frequently (**Fig 3E and 3F**). By contrast, ELAV/Hu-type binding sites were not enriched in the vicinity of included exons. A comprehensive summary of *de novo* motif searches are presented in **S6 Fig**. We obtained similar, but more exaggerated results, when we directly compared introns flanking excluded exons and included exons for differential motifs (**S6 Fig**). Visual inspection of upstream introns of the validated ELAV/Hu splicing targets confirm that introns flanking newly validated exon exclusion targets, such as *Medea* and *Axin*, and the recently recognition that *Fne* is an exquisitely sensitive exon exclusion target of Elav [44,45], tend to have far more matches to Elav consensus motifs in flanking introns than the newly validated exon inclusion targets (**Fig 3G**). Interestingly, the previously known exclusion target *arm* was the only validated targed that lacked a preponderence of consensus Elav consensus motifs, although it had other U-rich regions (**Fig 3G**).

In summary, we reveal that *Drosophila* ELAV/Hu proteins extensively reorganize alternative splicing. Our analyses depend on sufficiency tests that demonstrate similar capacities of multiple ELAV/Hu members, which led us to recognize endogenous functional overlap by Elav and Fne to specify the neural transcriptome. *Drosophila* ELAV/Hu RBPs appear to exclude cassette exons via cognate motifs in intronic regions flanking regulated exons. We also observe the mediate broad networks of exon inclusion, which do not bear comparable enrichment of ELAV/Hu binding sites. This might involve dispersed low frequency sites; indeed, we can find matches to Elav consensus motifs flanking validated exons (**Fig 3G**), even though these are not statistically enriched as with exon exclusion events. Alternatively, their processing may be assisted by an additional determinants, as suggested by the distinct nucleotide and motif content of their flanking introns (**S6 Fig**).

### Hierarchical activities of ELAV/Hu factors also drive global distal ALE splicing

A special class of regulated splicing occurs at alternative last exons (ALEs, **Fig 1**). As noted, two genes (*ewg* and *nrg*) were known to have Elav-dependent neural ALE splicing [41,49], and ectopic Elav/Fne/Rbp9 have common abilities to promote distal ALE switching of *nrg* [55]. To gain broader insight into regulation of ALE by ELAV/Hu factors, we exploited 3’-seq datasets from S2-GOF and L1-CNS-LOF conditions [44], which permit more precise quantification of 3’-isoform shifts than RNA-seq data [59].

**Fig 4:**
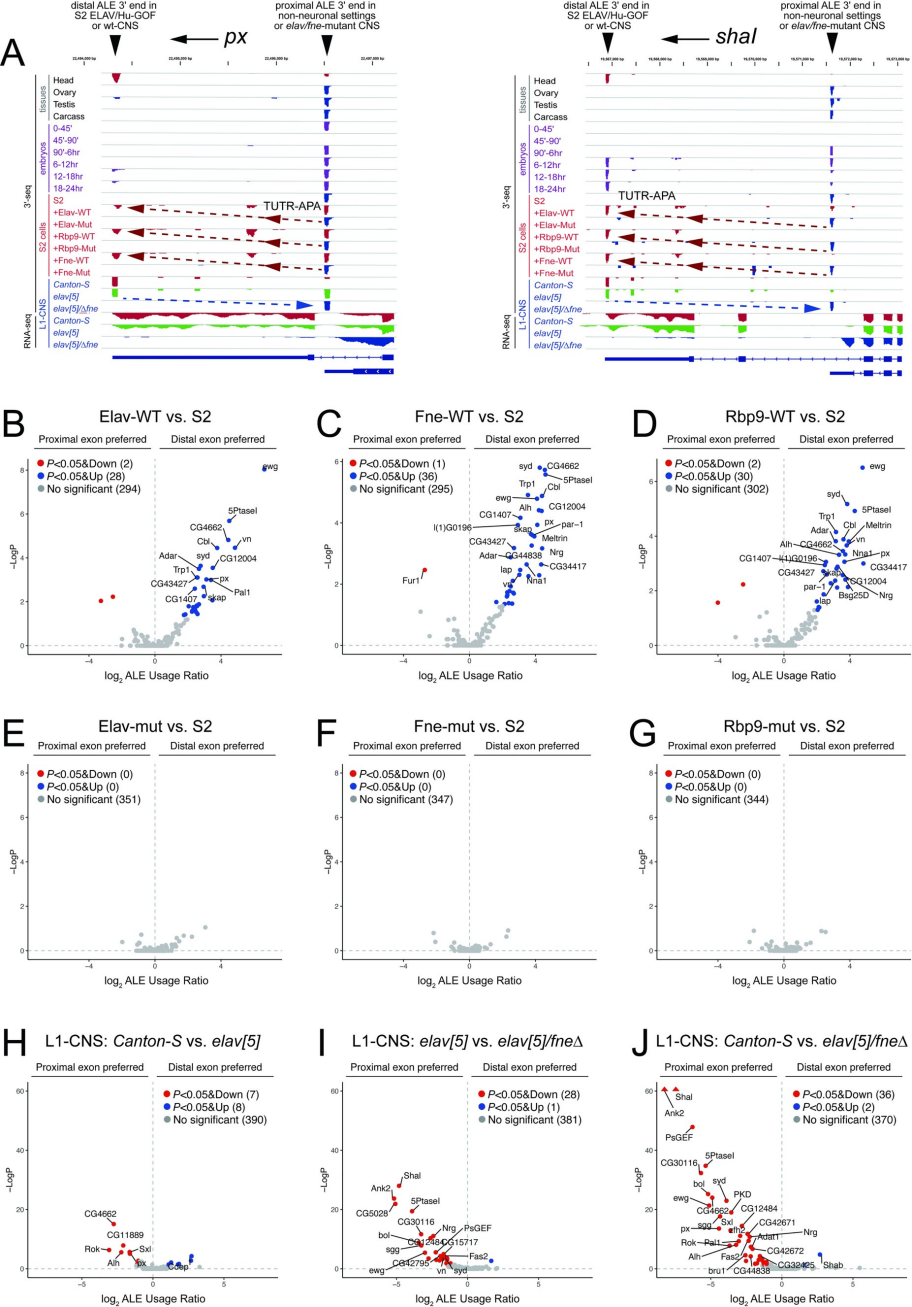
Overlapping activities of *Drosophila* ELAV/Hu RBPs control alternative last exon (ALE) splicing. (A) Examples of neural-specific distal ALE splice isoforms whose utilization exhibits necessity and sufficiency for ELAV/Hu RBPs. *px* and *ShaI* exemplify distal ALE isoforms that are (1) preferentially expressed in head compared to other tissues, (2) are developmentally induced during the timecourse of embryogenesis, (3) are induced in S2 cells upon transfection of wildtype Elav/Rbp9/Fne but not their RRM-mutant (Mut) counterparts, and (4) are expressed in dissected wildtype *Canton-S* and *elav[5]* null L1-CNS, but not in *elav[5]/Δfne* double mutant L1-CNS. Tracks are labeled as to 3’-seq or RNA-seq data. (B-D) Global analysis of misexpression of wildtype ELAV/Hu RBPs in S2 cells shows highly directional induction of distal ALE isoforms. (E-G) Misexpression of RRM-mutant ELAV/Hu RBPs in S2 cells does not alter ALE isoform usage. (H-I) Combined deletion of *elav* and *fne* causes a global reversion of distal-to-proximal ALE isoform usage switch in L1-CNS.

We illustrate newly recognized examples of these loci in **Figs 4A** and **S7**. In particular, *px* and *shaI* embody relevant expression and regulatory principles (**Fig 4A**). First, when sampling wildtype tissue data (head, ovary, testis, and carcass) or across a series of 6 timepoints that span embryonic development, we see that these genes preferentially or selectively express the distal ALE isoforms in the head or in late embryo stages when the nervous system has begun to differentiate. Second, comparing S2 cells that express wildtype or RRM-mutant ELAV/Hu factors, we see that all wildtype conditions and no mutant conditions are associated with ectopic induction of distal ALE isoforms. Third, we see that the distal ALE isoforms are present in wildtype and *elav* null L1-CNS, but revert to proximal ALE isoforms in *elav/fne* double mutant L1-CNS (**Fig 4A**).

Since rMATS does not evaluate alternative last exon (ALE) splicing, we utilized our 3’-seq analysis pipelines [60] to assess ALE switching. Notably, ectopic Elav/Fne/Rbp9 all induced global shifts in ALE splicing in S2 cells (**Fig 4B–4D**). These changes were quantitatively robust, affected a largely overlapping set of genes, and caused a directional shift towards distal 3’ UTR usage. In addition, these effects were fully dependent on their RNA-binding capacities, since their all 3xRRM-mutant versions were relatively inert (**Fig 4E–4G)**.

Based on this, we assessed the endogenous requirement of ELAV/Hu factors for distal ALE switching. Notably, Elav is required for neural distal ALE switching of *ewg* and *nrg* in photoreceptors, while *fne/rbp9* were reported to be dispensable for this process, either in photoreceptors or more generally in third instar larval or adult CNS [55]. However, ALE deployment has not been examined in first instar larval CNS. Perhaps surprisingly, we observed almost no ALE changes in these genes between control and *elav[5]* mutants (**Fig 4H**), even though the neurons in these samples have definitively been null for Elav for 12–18 hours. However, we observed substantial shifts towards proximal ALE isoform usage in *elav[5]/Δfne* double mutants; the differences were greater than when comparing *Canton-S* to *elav[5]* (**Fig 4I and 4J**). Overall, we frequently observe reciprocal behavior between the GOF and LOF datasets with respect to alteration of distal ALE isoform usage (**Figs 4** and **S7**), indicating that combined activity of ELAV/Hu RBPs plays a broad endogenous role to induce distal ALE isoforms in the larval CNS.

While this study was under review, Hilgers and colleagues also reported that *elav/fne* double mutant embryo RNA-seq data showed defects in the expression of a set of neural alternative last exons [45]. For reasons described above, whole embryo data are under-powered to determine these isoform changes. Moreover, 3’-seq data are superior to RNA-seq data for quantification of 3’ isoforms [60]. We compared the isoform usage changes in the *elav/fne* mutant embryo and the L1-CNS data, and broadly observe that ALE-APA events annotated uniquely from embryos (referred to as CDS-APA, [45]) exhibit minor changes at best, while annotations unique to our analysis exhibit robust changes and frequently have reciprocal processing in GOF data (**S8 Fig**).

**Fig 5:**
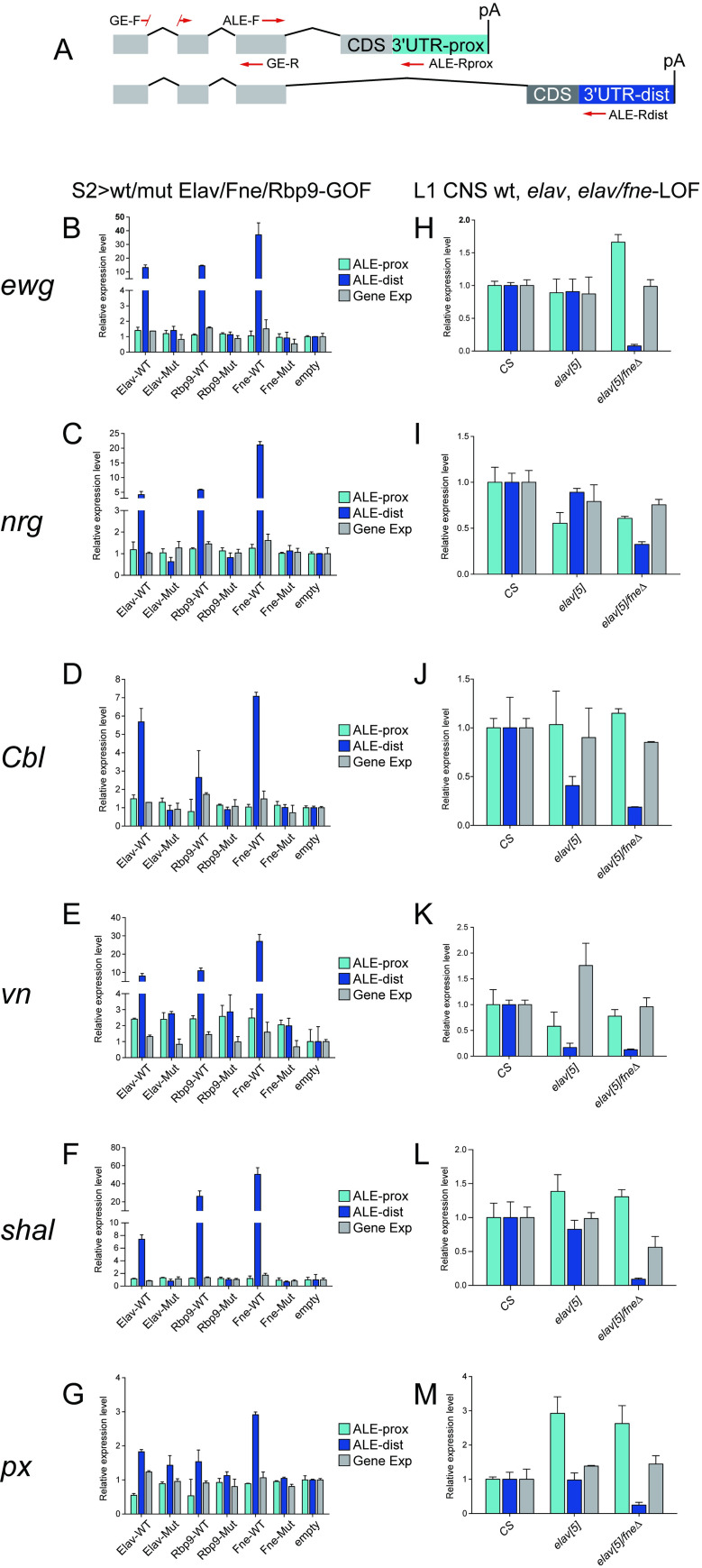
Experimental validation of distal ALE switching mediated by *Drosophila* ELAV/Hu RBPs. (A) Schematic of ALE isoforms and qPCR primers to assess gene expression (GE), proximal ALE isoform, and distal ALE isoform expression. Data were normalized to *rpl32*. (B-G) Assessment of 6 ALE targets in S2 cells upon mis-expression of wildtype (WT) or 3xRRM-mutant (Mut) forms of Elav, Rbp9 and Fne. All ELAV/Hu RBPs induce distal ALE isoforms, dependent on their RNA binding capacity, with relatively little effects on total locus expression. (H-M) Assessment of the same ALE targets in L1-CNS upon endogenous deletion of *elav* or both *elav/fne*. Certain targets exhibit sensitive distal ALE loss in single mutant conditions (e.g. *Cbl* and *vn*), but all of these loci exhibit decreased distal ALE isoform expression in the double mutant. All qPCR experiments were repeated twice, with each containing three replicates. Error bars are mean ± SD.

We validated these genomic data using quantitative rt-PCR for universal exon, proximal 3’ UTR and distal 3’ UTR amplicons of ALE loci (**Fig 5A**). With such assays, we could quantify changes in ALE isoforms as well as determine overall changes in gene expression. To validate our S2 samples, we selected additional neural-extension APA targets from our genomic data [44] and observed that tandem 3’ UTR extensions of *tai* and *ctp* were specifically induced upon expression of wildtype (but not 3xRRM-mutant) Elav/Fne/Rbp9 in S2 cells (**S9 Fig**). With these confirmations, we tested six ALE loci and observed that ectopic Elav/Rbp9/Fne all have RRM-dependent capacities to promote the accumulation of distal ALE isoforms (**Fig 5B–5G**). In general, these isoform changes do not substantially affect total gene expression levels. We observed opposite effects in L1 larvae (**Fig 5H–5M**). For the most part, single *elav[5]* null larvae did not exhibit ALE switching. This was particularly notable for *ewg* and *nrg*, which exhibit high dependency on Elav in the larval eye disc [41,49], but not in L1 larvae (**Fig 5H–5I**). Other ALE loci such as *shal* and *px* were also not affected in *elav* mutant L1, but we did find two loci (*Cbl* and *vn*) with selective loss of distal ALE isoforms in this background (**Fig 5J and 5K**). However, the effects in *elav/fne* double mutant L1 larvae were much more severe. With the exception of *vn*, whose strong loss in the single mutant did not provide room for enhancement, *Cbl* exhibited further loss of distal ALE expression in the double while the other four genes exhibit synthetic phenotypes in the double mutant (**Fig 5H–5M**).

Altogether, these GOF and LOF analyses broadly extend the role of ELAV/Hu factors in driving distal ALE splicing in *Drosophila* neurons. Moreover, they affirm that Fne plays a critical role to maintain neural ALE splicing when Elav is absent from larval CNS, just as it does with cassette exon splicing (**Figs 2 and 3**). The complete ALE splicing analyses are provided in **S2 Table**.

### ELAV/Hu RBPs induce distal ALE usage by inhibiting pA site usage of internal isoforms

We investigated mechanisms for how ELAV/Hu family RBPs induce distal alternative last exon splicing (**Fig 1**). In principle, this could be regulated at the level of splicing into the proximal alternative exon (ALE), or by altering the stability of the different isoforms. However, in the case of *ewg* [40] and *nrg* [41], Elav binds downstream of cleavage sites for proximal ALE isoforms and inhibits their usage ("pA site bypass"), thereby permitting further transcription and splicing into the downstream ALE isoform. Notably, we found broad evidence for an analogous model as to how ELAV/Hu proteins implement neural 3’ UTR lengthening. Proximal pA sites that are bypassed in the nervous system exhibit strong downstream enrichment of ELAV/Hu binding sites [44]; concurrent studies support this notion [45]. Therefore, we tested if these principles might apply more broadly to other ALE targets, and across other members of the ELAV/Hu RBP family.

**Fig 6:**
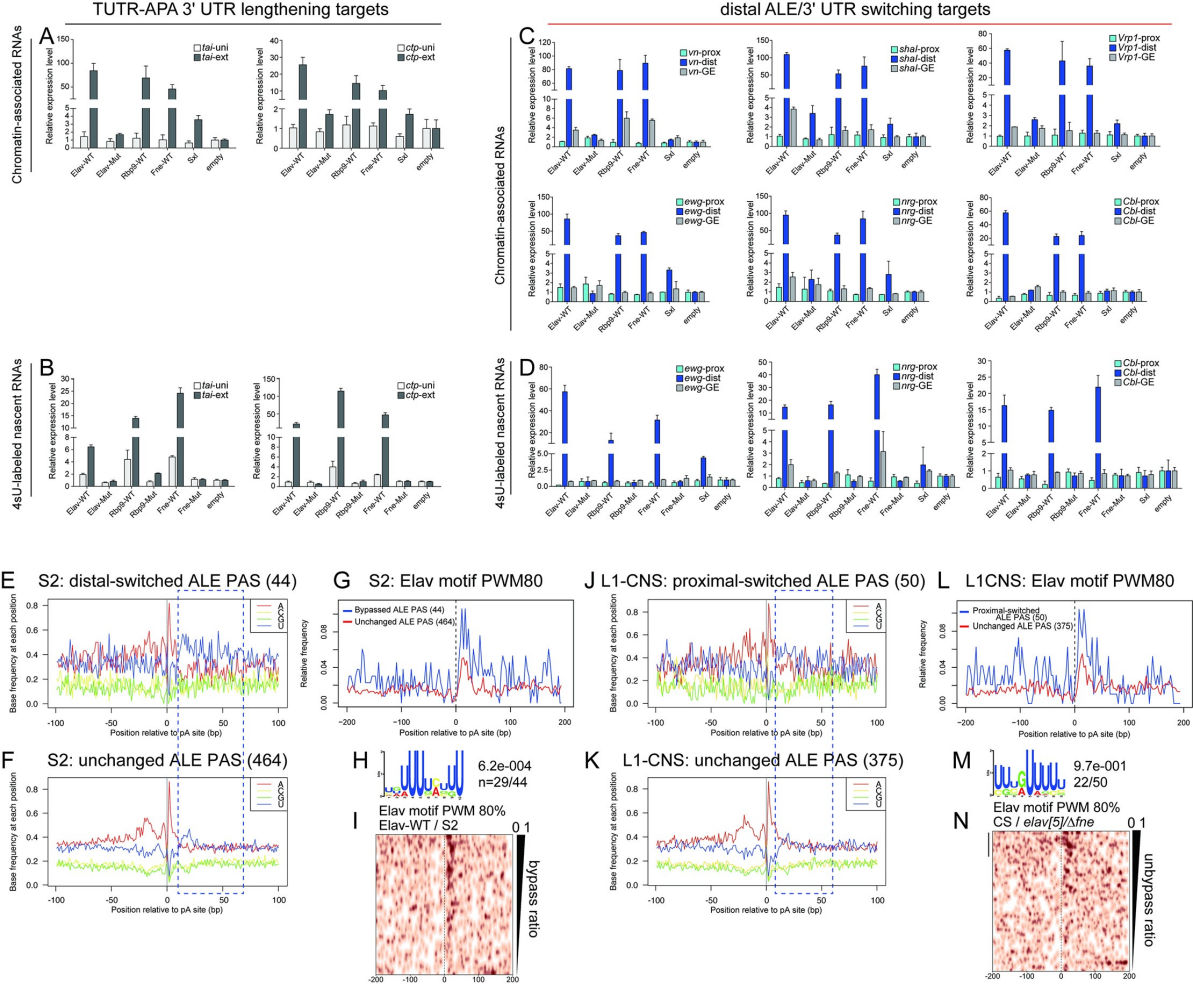
Mechanism of ALE-APA control by ELAV/Hu RBPs is shared with TUTR-APA targets. (A-B) Validation of new terminal 3’ UTR-APA (TUTR-APA) targets in the S2-GOF system. The 3’ UTR extensions of *tai* and *ctp* can be detected in chromatin-associated fractions (A) and in nascent transcripts isolated using 4sU labeling (B). (C) Distal ALE switching induced by GOF of ELAV/Hu RBPs also occurs in chromatin-associated fractions, but is not detected in RNA binding-defective Elav or with the homolog RBP Sxl. (D) Distal ALE switching induced by GOF of ELAV/Hu RBPs is detected in newly-synthesized transcripts. (E-I) Nucleotide and motif analysis of regions surrounding proximal ALE polyadenylation (pA) sites in S2 cells. (E-F) Nucleotide frequency of ALE cleavage sites that are bypassed by ELAV/Hu RBP-GOF (E) or that are unchanged (F) shows enrichment of downstream uridine amongst regulated loci. (G) Elav binding sites (80% match to position weight matrix, PWM80) are enriched downstream of regulated proximal ALE pA sites. (H) De novo search shows the Elav binding site is the most frequent and most significant motif downstream of regulated ALE pA sites. (I) Elav binding sites are correlated with the frequency of bypass as measured by distal isoform induction in the presence of ectopic Elav in S2 cells. (J-N) Nucleotide and motif analysis of regions surrounding proximal ALE pA sites in L1-CNS. (J-K) Nucleotide frequency of ALE cleavage sites that are switched from distal to proximal usage by *elav/fne-*LOF (J) or that are unchanged (K) shows enrichment of downstream uridine amongst regulated loci. (L) Elav binding sites are enriched downstream of regulated proximal ALE pA sites. (M) De novo search shows the Elav binding site is the most frequent and most significant motif downstream of endogenously regulated ALE pA sites in L1-CNS. (N) Elav binding sites correlate with distal-to-proximal ALE isoform switching in *elav/fne* mutant L1-CNS.

We used the controlled S2 cell system to assess whether ELAV/Hu-mediated distal ALE switching occurred in subcellular and/or temporal contexts that were consistent with the pA site bypass model. For this purpose, we compared the levels of proximal and distal ALE isoforms with common amplicons for a panel of loci, in response to all three wildtype ELAV/Hu RBPs, 3xRRM-mut variant(s), or Sxl as a control U-rich binding RBP. For comparison, we performed additional assays of newly-validated TUTR-APA 3’ UTR lengthening targets *tai* and *ctp* (**S9 Fig**) and confirmed that ELAV/Hu RBP-induced 3’ UTR extension occurs in chromatin-associated transcripts (**Fig 6A**) and in newly-synthesized RNAs isolated by 4sU labeling (**Fig 6B**). We obtained reliably similar and specific induction of a panel of distal ALE isoforms by wildtype ELAV/Hu RBPs (**Fig 6C and 6D**), indicating similar features of molecular regulation for 3’ UTR lengthening and distal ALE switching.

We then examined if there were characteristic motif features at the 3’ termini of genes subject to ELAV/Hu-induced distal ALE switching. We first examined PAS, and classified them as to whether they were at the termini of the internal or the distal ALE isoforms, or whether they were internal to 3’ UTRs (**S10A Fig**). As a reference, we analyzed single-end S2 cell genes, which are expected to harbor strong PAS. We represent the frequency of consensus PAS motifs 10–30 nt upstream of the mapped cleavage sites from 3’-seq data in stacked bar plots. We compared this to PAS derived from two types of S2-expressed ALE gene models, those that collectively exhibited distal ALE usage upon gain-of-function of Elav/Fne/Rbp9 (38 genes, 44 3’ UTRs) and those that were insensitive to their action (367 genes, 464 3’ UTRs). We observed that the PAS quality of internal ALE 3’ termini was substantially lower than constitutive 3’ ends, implying that they are preferentially bypassable (**S10B Fig**). However, there was no substantial difference between regulated and non-regulated ALE genes.

We next examined for sequence characteristics in the vicinity of cleavage sites. We plotted the nucleotide distribution +/-100 nt of cleavage sites mapped by 3’-seq data, for internal ALE termini that were or were not sensitive to ELAV/Hu factors in S2 cells. Although the pattern was more noisy for the smaller ELAV/Hu-sensitive dataset, these plots indicated enhanced frequency of uridine downstream of their cleavage sites, compared to the control set (**Fig 6E and 6F**). This disparity was emphasized by plotting the uridine frequency downstream of cleavage sites between these sets of internal ALE termini (**Fig 6**). Thus, although a U/GU-rich sequence downstream of pA sites generally contributes to cleavage by recruiting CstF, internal ALE termini that can be bypassed in the presence of ELAV/Hu factors exhibit distinct U-rich downstream context.

We followed this up using *de novo* motif searches. Notably, the top significant and most frequent hit (present in 2/3 of loci) downstream of internal ALE termini that were bypassed by ELAV/Hu factors was a U-rich motif that closely resembled the consensus for ELAV/Hu RBPs (**Fig 6H**). Such a motif was either not found in other PAS categories (e.g., downstream of pA sites that were internal to 3’ UTRs of these genes, or downstream of the distal ALE termini of these genes), or was found at low frequency (9–12%) and with a different U-rich features (e.g., downstream of some classes of termini in genes unaffected by ELAV/Hu factors) (**S11 Fig**). This motif is very similar to the motif recovered at cassette exons that are excluded by ELAV/Hu RBPs, from both GOF and LOF datasets (**Fig 3E and 3F**).

We performed similar analyses using larval CNS genes whose distal ALE usage was suppressed in *elav/fne* mutants compared to from *Canton-S* (wt). Here, we focused on genes expressed in CNS, which are only partially overlapping with S2-expressed genes, and thus reflect an independent analysis. The overall picture was quite similar. All CNS genes that are processed into multiple ALE isoforms exhibit somewhat weaker PAS at the termini of the internal ALE isoforms (**S10D Fig**), but the major characteristic of ALE loci dependent on endogenous Elav/Fne is the presence of elevated uridine content downstream of pA sites (**Fig 6J and 6K**). Again, *de novo* motif searches show a high-frequency (44%) of U-rich motifs that closely resemble Elav binding sites downstream of internal ALE termini that are aberrantly expressed in *elav/fne* mutants (**Fig 6L and 6M**), whereas the frequency of such U-rich motifs downstream of other internal ALE termini is much lower (13%) (**S11 Fig**). The enrichment of such motifs is greatest downstream of proximal ALE 3’ termini that are misexpressed in *elav/fne* mutants (**Fig 6N**), consistent with the notion that the combined activities of these ELAV/Hu RBPs is needed to switch these to distal last exon isoforms.

Taken together, these features extend the strategy of Elav-mediated pA bypass for distal ALE splice isoform switching, from *ewg* and *nrg* to many dozens of *Drosophila* loci. Moreover, we broaden this from Elav, thought to be the only nuclear ELAV/Hu family member in *Drosophila*, to other members. We show that in ectopic settings, all three members are able to drive distal ALE isoform usage by inducing downstream nascent transcription in the chromatin fraction, following pA site inhibition. Moreover, our comparison of single *elav* and double *elav/fne* mutants indicate that Fne plays a major role in controlling ALE splicing.

### Generation of Elav CLIP maps from S2 cells

To gain further insights into the nature of direct regulatory networks controlled by ELAV/Hu factors, we investigated cross-linking and immunoprecipitation-sequencing (CLIP-seq) data. While our study was under review, Hilgers and colleagues reported Elav CLIP-seq data from fly heads [45], a relevant endogenous setting. Although it was concluded these data show Elav is directly and specifically bound to its APA and splicing targets [45], examination of the data prompted a more circumspect view. In particular, while there is local Elav binding at regulated exons and pA sites, by (1) inspecting the total mapped reads instead of displaying binarized (0 vs 1) peak calls and by (2) examining larger windows of gene models, the head Elav CLIP signals are not generally very specific (**S3 and S8 Figs**). In addition, as no input library was reported, and the total number of genes recovered in the Elav CLIP dataset is large (>3000 genes at 5CPM, >1200 genes at 40 CPM, **S3 Table**), it was unclear how to set cutoffs.

**Fig 7:**
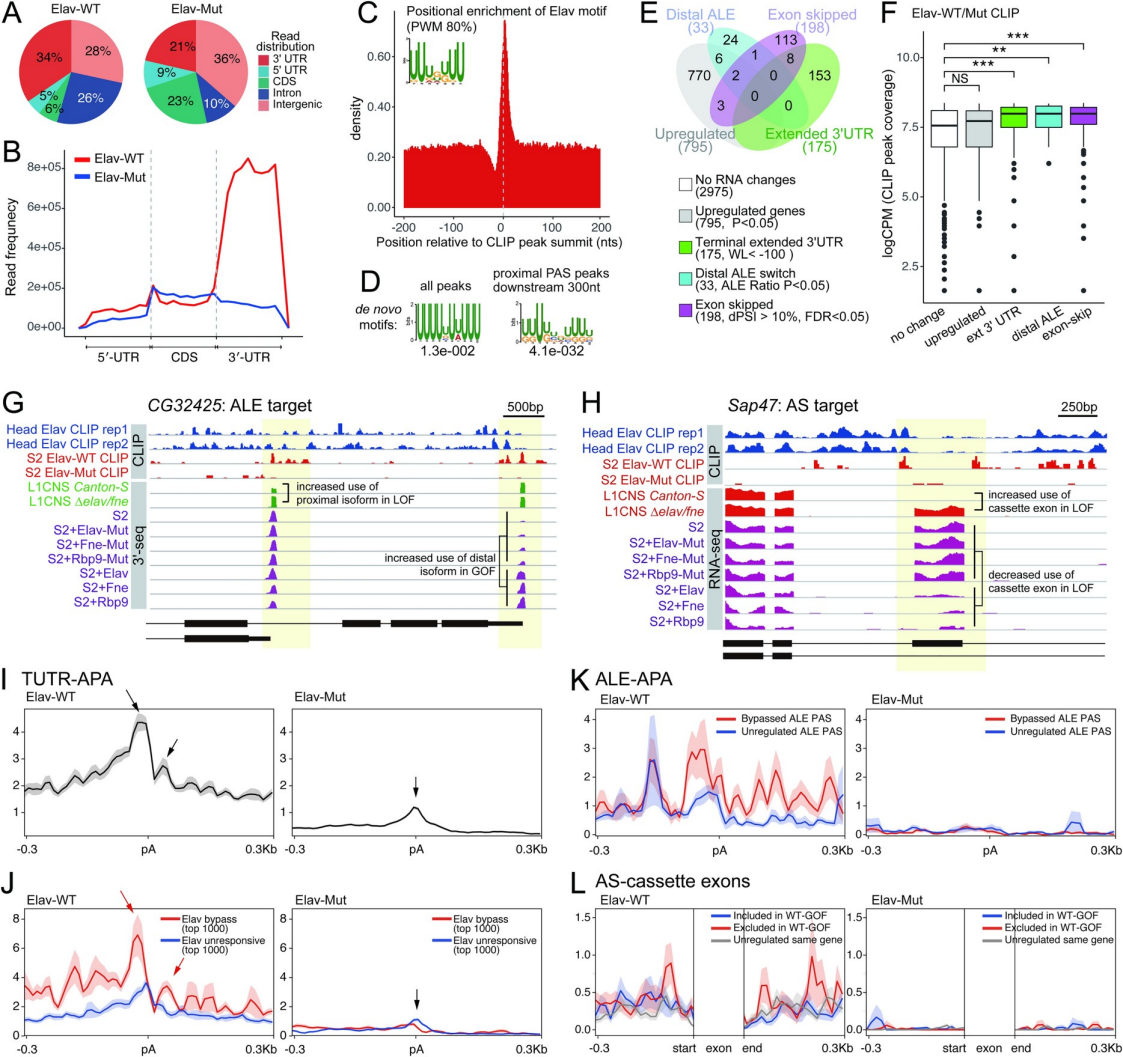
Genomic analysis of RRM-dependent Elav binding in S2 cells. (A) We used BrdU-CLIP to profile wildtype (wt) and 3xRRM-mut (Mut) Elav expressed in S2 cells. Pie charts show the genomic distribution of WT and 3xRRM-mut (Mut) Elav CLIP-seq reads. (B) Metagene profiles of WT and Mut Elav CLIP-seq read distribution across the transcriptome of S2 cells. (C) Positional distribution of the Elav motif within CLIP peak clusters. Sequences that match ≥80% PWM are enriched at CLIP peak summit. (D) *De novo* sequence motifs enriched within the transcriptome (left) or 300 nt downstream of proximal pA sites from CLIP peak clusters. (E) Venn diagram of four classes of genes that are altered by expression of wildtype-ELAV/Hu RBPs shows they are mostly non-overlapping. (F) CLIP peak coverage of each of the four categorized genes. Coverage of CLIP peaks spanning in the whole gene body including introns are defined as the coverage of a give gene. Genes that are upregulated in the presence of ELAV/Hu factors exhibit similar Elav-CLIP binding as bound genes lacking RNA changes, but 3’ UTR extension, distal ALE switching, and exon-skipping targets all show elevated binding of Elav in aggregate. (G-H) IGV browser views of representative Elav/Hu-regulated ALE (H) and cassette exon exclusion (H) targets. (I-L) Metagene profiles of Elav-WT and Elav-Mut CLIP clusters in the vicinity of regulated isoform sites. (I) All pA sites in terminal 3’ UTRs. (J) Segregation of pA sites that are most or least responsive to ectopic ELAV/Hu proteins. (K) Analysis of Elav binding at ALE 3’ ends, segregated by sites that are bypassed or unaffected by ectopic ELAV/Hu proteins. (L) CLIP signals from 300 nt upstream of the 3’-splice site and downstream of the 5’-splice site of alternatively spliced cassette exons regulated by ELAV/Hu RBPs. These are segregated as to exon exclusion or inclusion, and all other exons of the same genes are plotted as controls. Metaplots in I-L show average profiles and standard errors for the indicated sets of loci.

We therefore generated Elav CLIP-seq data from S2 cells, where we have shown that ectopic Elav induces global cassette exon and ALE splicing changes (**Figs 2–6**), as well as global APA and gene expression changes [44]. For this purpose, we compared CLIP profiles from Elav-wt with its 3x-RRM mutant (mut) counterpart, which is relatively inert but accumulates to a comparable level (**S4 Fig**). The overall binding of Elav-wt was strongly biased to 3’ UTRs, the known location of ELAV/Hu factors, while Elav-mut did not exhibit such bias (**Figs 7A and 7B** and **S12**). Directed searches showed that Elav-wt peaks were enriched for consensus Elav/Hu binding sites (**Fig 7C**). Moreover, *de novo* motif searches recovered statistically significant enrichment of ELAV/Hu-like sites genomewide amongst all peaks, but these comprised by far the top motif when restricting the search to local regions downstream of proximal pA sites (**Figs 7D** and **S13**). By contrast, no such U-rich motif could be found in Elav-mut peaks (**S13 Fig**). Thus, the Elav CLIP map yields information on direct binding of Elav to the transcriptome.

### Elav CLIP shows broad occupancy but supports its roles in generating isoform diversity

We sought to use the Elav CLIP data to connect Elav to direct regulation of targets. Although this is a misexpression setting, a substantial fraction of these changes are reciprocal to loss-of-function of endogenous ELAV/Hu factors. Still, we were cautious to interpret our Elav-CLIP data, since similar to the head Elav-CLIP map [45], there are up to thousands of loci captured in our S2 Elav-CLIP map, depending on the cutoff applied (**S3 Table**). Unlike the head data, however, we can infer RRM-dependent interactions, since there were several-fold more genes captured by Elav-wt than by Elav-mut at different cutoffs (**S3 Table**).

We took the opportunity to assess binding across different categories of targets. We find loci that are upregulated, switch to exon skipped isoforms, switch to distal alternative last exons, or generate extended terminal 3’ UTRs, comprise largely non-overlapping gene sets (**Fig 7E**). We analyzed the distribution of Elav CLIP binding to these groups of genes, compared to other loci with Elav binding that otherwise had no RNA changes. Interestingly, although mammalian ELAV/Hu RBPs are involved in RNA stabilization [35,61,62], we find no difference in the Elav occupancy of upregulated genes and ones with no RNA changes. By contrast, there was statistically significant elevation of Elav CLIP signals on the groups of targets that underwent exon skipping, distal ALE switching, and 3’ UTR extension (**Fig 7F**). While we cannot rule out that a subset of Elav CLIP targets are artifactual due to misexpression, or that some of these may mediate other regulatory outputs such as subcellular relocalization of translational regulation, these data support the notion that Elav binding is associated with alternative mRNA processing.

At the same time, these CLIP data point to broad association of Elav to the transcriptome, and the strong enrichment in 3’ UTRs suggests binding relevant to mature mRNAs and not to nascent mRNA processing (as inferred to by our mechanistic tests). We may expect binding to nascent mRNAs to be transient, while the dominant localization of Elav CLIP signals to 3’ UTRs, and to a lower extent to exons, masks potential signals that might exist in nascent transcripts. In the concurrent study of Elav head CLIP-seq, the exonic data was masked out to show flanking intronic data [45].

Inspection of individual loci suggested our S2 Elav-CLIP data showed more specificity than the head Elav-CLIP data (**S3 and S8 Figs**). In some cases, this reveals specific local binding in the vicinity of alternative isoforms. For example, we could identify loci with RRM-dependent Elav-CLIP signals in S2 cells downstream of certain ELAV/Hu-bypassed ALE 3’ ends (**Fig 7G**) or flanking certain cassette exons (**Fig 7H**), while corresponding head Elav-CLIP data [45] was broad across nearby exons and introns. However, neither dataset showed exclusively focal binding around regulated sites of mRNA processing. Since we previously annotated over one thousand ELAV/Hu binding sites in 3’ UTRs that are deeply conserved across Drosophilid species [60], and even a minor amount of cytoplasmic Elav proteins may be expected to generate stable complexes with mature mRNAs (**S4 Fig**). Nevertheless, by meta-analysis of the different classes of regulated loci, we could observe distinctions in the aggregate binding of Elav that correlate with mRNA processing.

First, we observe that Elav exhibited elevated binding to transcript termini, as has been observed in mammals [63]. However, further inspection shows a local peak just downstream of pA sites (**Fig 7I**), corresponding to the location of ELAV/Hu binding sites that we recently reported [44]. Although the enrichment is mild, it is not found in corresponding Elav-mut CLIP data, which instead shows a minor signal just above background centered on cleavage sites. It is possible that this is an artifact of a minor population of pA sites that happen to be prone to internal priming, since by far the top motif in Elav-mut CLIP data is a poly-A sequence.

To test if these biased patterns of Elav occupancy corresponded to genes undergoing APA, we plotted Elav occupancy at the subsets of pA sites that were most subject to bypass in the presence of ectopic Elav, or that were most resistant to altered usage. This analysis showed that Elav was indeed bound more highly just upstream and downstream of highly-bypassed cleavage sites, and that the profile was distinct at non-responsive genes (**Fig 7J**). Moreover, Elav-mut exhibited a minor peak centered on pA sites of non-responsive genes, confirming it is likely artifactual signal. These data indicate that while bulk Elav CLIP signals are distributed throughout 3’ UTRs, they can provide insights into its roles in mRNA processing.

Second, when examining genes undergoing distal alternative exon switching, compared to genes that do not respond to Elav, we observe local enrichment of Elav CLIP signals that are fully dependent on its RNA-binding capacity (**Fig 7K**). This is consistent with the notion that local binding of Elav is involved in suppressing pA usage at 3’ cleavage at these proximal last exons, to promote switching to distal last exons.

Third, we examined genes undergoing alternative splicing in the present of ectopic Elav. As in the related study [45], we masked out the central exons to better focus on intronic signals.

We compared introns flanking exons that were excluded or included in response to Elav, with those flanking unregulated exons of those genes as matched controls. We observed a moderate increase in Elav occupancy specifically in the introns that were excluded (**Fig 7L**). This was consistent with our *de novo* motif analysis showing enrichment of ELAV/Hu binding sites only surrounding exons that were excluded, but not included, in ectopic and endogenous manipulations of ELAV/Hu RBPs (**Fig 3E and 3F**).

Altogether, these CLIP data suggest potentially broad transcriptome association by ectopic Elav, which seems in line with an endogenous Elav CLIP dataset from heads [45]. However, our RRM-dependent Elav CLIP data also support our notion that local binding of Elav directly contributes to generation of isoform diversity of diverse classes of mRNA processing targets, as implied by the local enrichment of ELAV/Hu binding at these regulated isoforms.

### Evidence that neural programs of mammalian distal ALE splicing and 3’ UTR lengthening are coordinated by ELAV/Hu RBPs

Our recognition that distinctive programs of neural ALE and APA are both mediated by ELAV/Hu factors in *Drosophila* inspired us to search for evidence of analogous regulatory programs during mammalian neural differentiation. To do so, we took advantage of recent directed human iPSC-neuron directed differentiation datasets [64]. These analyses comprise 9 timepoints including 3 stages of iPSC culture in "accelerated dorsal" media (days 2, 6 and 9), the neural precursor cell (NPC) stage (day 15), the neural rosette stage (21), and several timepoints following neural specification (days 49, 63 and 77), during which cells were co-cultured with rat astrocytes to facilitate terminal maturation.

**Fig 8:**
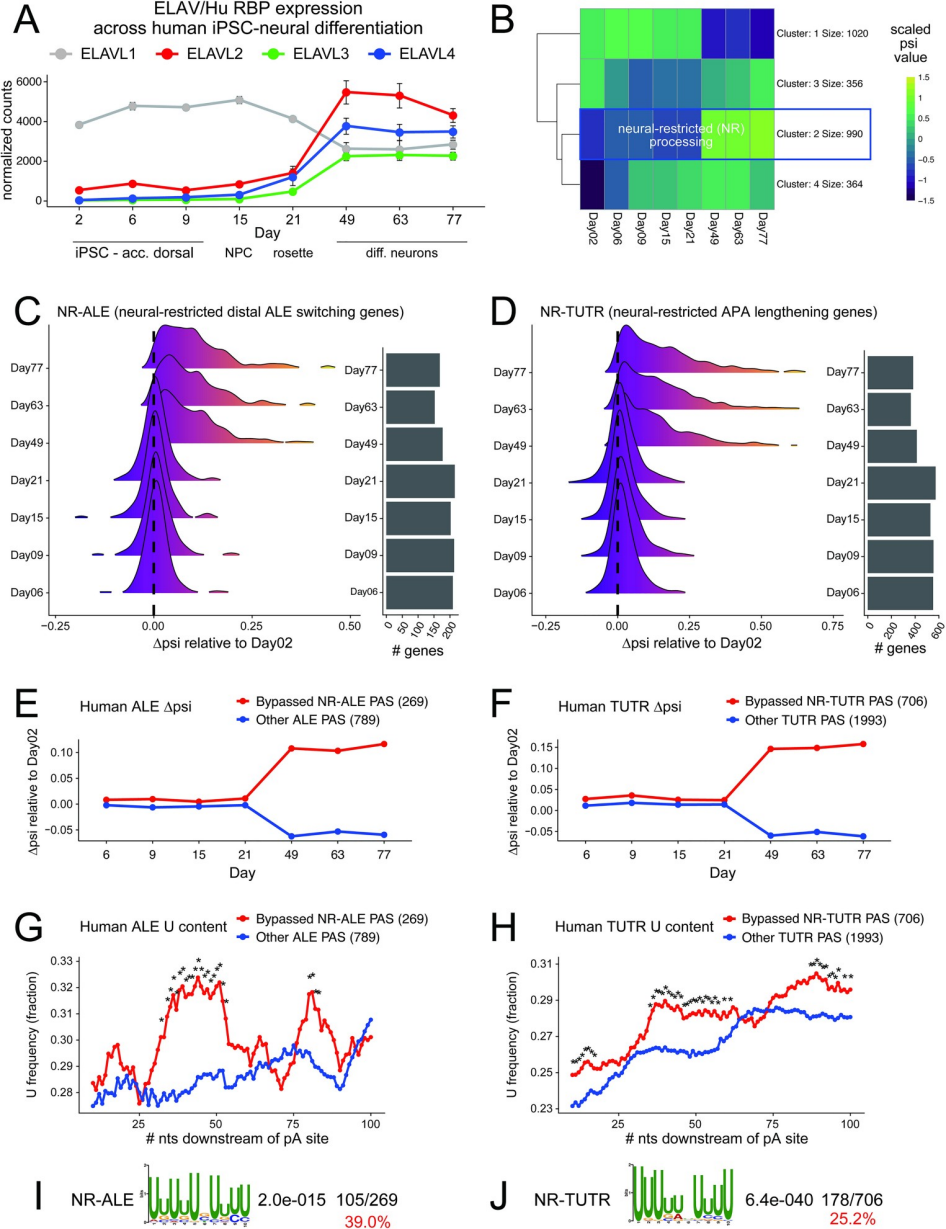
Evidence that mammalian neural ELAV/Hu RBPs are involved in distal ALE switching and 3’ UTR lengthening in neurons. (A) Expression of ELAV/Hu RBP members across a timecourse of directed differentiation of human iPSCs into neurons [64]. ELAVL1 (HuR) is ubiquitous and is downregulated upon neuronal specification, while ELAVL2-4 (HuB-D) are upregulated upon neuronal commitment. (B) K-means clustering identifies a cohort of genes with monotonically increasing *ψ* across the neuronal differentiation timecourse, i.e. genes with neural-restricted (NR) alternative processing. (C-D) Collective behavior of NR-ALE (C) and NR-APA (D) genes across the neuronal differentiation timecourse. (E-F) Average behavior of NR-ALE (E) and NR-APA (F) genes across the neuronal differentiation timecourse. (G-H) Both NR-ALE (G) and NR-APA (H) genes specifically exhibit enrichment of uridine downstream of polyadenylation (pA) sites that are bypassed in neurons, compared to pA sites in other ALE and TUTR genes expressed in neurons. (I-J) The 100 nt regions downstream of NR-ALE (I) and NR-TUTR pA sites bypassed in neurons are highly enriched for ELAV/Hu RBP binding sites.

Of the four mammalian ELAV/Hu members, ELAVL1/HuR is ubiquitously expressed, while ELAVL2-4/HuB-D are enriched in neurons. Accordingly, during iPSC->neuronal differentiation, *ELAVL1* transcripts are detected throughout, but are downregulated during the transition from neural rosettes to neurons. Concomitant with this, *ELAVL2/3/4* are all upregulated at this same transition, as neurons are first specified (**Fig 8A**).

To quantify APA across the neural differentiation time course, we used LABRAT (https://github.com/TaliaferroLab/LABRAT). LABRAT takes in RNAseq data and assigns psi (*ψ*) values to genes with *ψ* = 0 indicating exclusive usage of gene-proximal (upstream) polyadenylation sites and *ψ* = 1 indicating exclusive usage of gene-distal (downstream) polyadenylation sites. We limited our analysis to genes that contained only two polyadenylation sites. With the neural differentiation time course, we used k-means clustering to identify genes with steadily increasing *ψ* values throughout the differentiation. We termed these genes as having neural-restricted (NR) alternative processing. These include 706 genes with shifts towards longer 3’ UTRs within terminal 3’ UTRs (TUTR), 269 genes with shifts towards distal alternative last exons (ALE) (**Fig 8B)**. From these, we defined two substantial classes of NR genes, which directionally express more distal ALEs or lengthened TUTR isoforms (**Fig 8C and 8D**). Both classes of NR-ALE and NR-APA genes exhibited isoform shifts during the transition from neural rosettes into post-mitotic neurons, concordant with upregulation of *ELAVL2/3/4*. The remainder of genes that were expressed at the end of the timecourse (**Fig 8B**) were used as controls, which collectively exhibit unchanged or lower *ψ* values in differentiated neurons compared to undifferentiated samples (**Figs 8E and 8F** and **S14**). The full NR-ALE and NR-APA data are provided in **S4 Table**.

We analyzed the sequences downstream of NR-ALE and NR-TUTR proximal polyadenylation sites, and found that both exhibited significantly higher U-content than their respective controls (**Fig 8G and 8H**). This was likely attributable to binding by neuronal ELAV/Hu proteins, because de novo motif analysis revealed high frequency and highly significant occurrences of characteristic ELAV/Hu binding sites downstream of both NR-ALE and NR-TUTR proximal polyadenylation sites (**Fig 8I and 8J**).

Altogether, these bioinformatic analyses provide evidence for coordination of distal ALE switching and terminal 3’ UTR lengthening in mammalian neurons by neuronal ELAV/Hu RBPs, indicating that these two broad strategies of mRNA processing control in neurons are conserved across metazoans.

## Discussion

### Combined activities of *Drosophila* ELAV/Hu RBPs specify the neural splicing landscape

Mammalian ELAV/Hu RBPs have been extensively connected to alternative splicing of cassette exons [19,46–48,50], but only to selected APA events [39,65,66]. In contrast, only a handful of *Drosophila* genes were known to be alternative splicing targets of Elav [40,42,43,67], of which only two loci (*Dscam1* and *arm*) harbor regulated cassette exons. Thus, it was unclear to what extent there are conserved utilities of this RBP family in mRNA processing.

Here, we show that all three ELAV/Hu members specifying hundreds of alternative splicing events. We show endogenous relevance, by demonstrating that dual deletion of *elav* and *fne* causes reciprocal changes to splice isoform accumulation. Notably, we reveal the endogenous breadth of splicing control by ELAV/Hu RBPs by analyzing dissected larval CNS, which contains more mature neurons than embryos and also removes the expression of non-neuronal isoforms outside of the nervous system from consideration. In particular, *elav* null L1-CNS has only mild effects on alternative splicing, despite its lethality, and analysis of *fne* nulls showed no effects on specific targets. Thus, the combined activity of ELAV/Hu RBPs, likely involving a hierarchial suppression of Fne nuclear localization via exon-exclusion of *fne* splicing by Elav [44,45], is critical to broadly determine neuronal mRNA isoforms.

Until now, our evidence for roles of Rbp9 in mRNA processing is based on ectopic expression. Even though *Drosophila* ELAV/Hu RBPs exhibit distinct subcellular preferences, all of them exhibit similar binding capacities *in vitro* [55], and have overlapping regulatory capacities in ectopic assays. Since we could not obtain triple mutant larvae of *Drosophila* ELAV/Hu members, we were not able to assay nervous system devoid of this RBP family. This may require creative conditional genetics to achieve the requisite conditions, especially in pupal and/or adult stages, when Rbp9 is expressed at much higher levels in the nervous system [44].

We observe substantial differences in the flanking intronic content of exon classes that are regulated ELAV/Hu RBPs. Their exclusion targets are substantially enriched for characteristic U-rich ELAV/Hu binding motifs, and have elevated Elav-CLIP signal, but such features were not observed with their inclusion targets. In general, little is known of the mechanism of splicing control by ELAV/Hu RBPs. In mammals, exclusion of a *Fas* cassette exon by HuR was reported to involve competition with U2AF65 at the upstream 3’ splice site [50]. A competition model is potentially consistent with our fly data, since we observe substantially higher density of ELAV/Hu RBP motifs upstream of excluded exons (**Fig 2L and 2M**). However, we also observe enrichment of ELAV/Hu RBP motifs downstream, although to a lesser extent. For exons that are preferentially included in the presence of ELAV/Hu members, they might still depend on binding that is below the sensitivity of these analyses. Another possibility is that these exons might involve additional regulatory factors, which is hinted at by enrichment for A-rich motifs located downstream of regulated exons. We note that PABP, PABP2 (PABPN1), ZC3H14/dNab2, and hnRNP-Q (Syncrip) proteins associate with qualitatively similar A-rich motifs [58,68,69], and include known neuronal splicing regulators. The discovery of extensive ELAV/Hu-mediated cassette exon targets, including the finding that individual ELAV/Hu proteins can robustly induce exon exclusion and inclusion in an ectopic context, provides a framework for future mechanistic dissection.

### Broad mechanistic analogy for generation of ALE splicing and terminal APA isoforms

Many studies in the literature have treated ALEs and tandem UTRs separately, since ALEs may be regulated by splicing while tandem UTRs are only regulated by alternative polyadenylation (**Fig 1**). Nevertheless, distal ALE and downstream tandem APA usage are correlated in mammals, with directionality toward more distal/longer isoforms in neurons [26]. The underlying mechanisms have not been specifically defined. It is known that telescripting via U1 snRNP suppresses premature 3’-end cleavage and polyadenylation [70]. While this can occur in intronic regions and terminal 3’ UTRs, the dominant usage of this mechanism seems to be for U1 to inhibit the usage of cryptic PAS that are especially abundant within long introns, and U1/telescripting has not yet been shown to have a broad impact on endogenous tissue-specific implementation of 3’ isoforms.

*Drosophila* Elav was linked to both isoform regulatory programs, since it was originally shown to promote distal ALE switching by suppressing 3’ end usage of proximal internal last exons at *ewg* and *nrg* [40,67] and later shown to mediate neuronal 3’ extension of select loci [38]. Likewise, regulation of APA was shown for all four Hu proteins in suppressing an intronic polyA site in the *calcitonin/CGRP* gene and HuR autoregulates by APA [39,65,66]. In addition, HuR regulates 3’-end processing of several membrane proteins [71]. This individual cases set the possibility that ELAV/Hu RBPs may coregulate these programs.

In concurrent work, we established that the three *Drosophila* ELAV/Hu members (Elav/Fne/Rbp9) are individually sufficient to induce the neural extended 3’ UTR landscape, and that endogenous overlapping activities of *Drosophila* Elav and its paralog Fne are critical to determine the extended 3’ UTR landscape of the larval CNS [44], as also shown in the embryo [45]. In this study, we extend this to reveal broad catalogs of directional ALE isoform switches by ELAV/Hu factors. Using mechanistic tests and genomic analyses of *de novo* motif and RRM-dependent Elav CLIP maps we are now able to unify the rationale for distinct neuronal mRNA processing programs. In particular, *Drosophila* ELAV/Hu RBPs are necessary and sufficient to specify broad switching to distal alternative last exons, analogous to broad lengthening of terminal 3’ UTRs via usage of distal pA sites [44,45]. In both settings, ELAV/Hu RBPs suppress proximal pA sites via downstream U-rich sequences/ELAV motifs downstream of cleavage sites, and promote distal isoform usage by acting within newly-synthesized, chromatin-associated transcripts. Since we also find that ELAV/Hu proteins are broadly involved in exon exclusion, via overt enrichment of their sites near regulated exons, we suggest broad analogies for ELAV/Hu RBPs to promote isoform diversity by suppression of processing sites used outside of the nervous system.

Importantly, we suggest that similar regulatory rationale applies to the implementation of both neuronal ALE and APA in mammalian neurons. In particular, we provide evidence that ELAV/Hu RBPs are poised to regulate both classes of 3’ ends using similar mechanisms (i.e. polyA bypass mediated through U-rich sequences). Mammalian ELAV/Hu factors are well-known to mediate diverse regulatory outputs, ranging from mRNA stability [35,61,62] and translation [72–74], to splicing [50,75] and terminal APA regulation of selected loci [39,65,66]. However, they are not yet documented to have broad roles in directional selection of alternative last exons or pA sites within terminal 3’ UTRs. Our genomic analyses (**Fig 8**) now lend strong support to this notion.

Given that Elav paralogs have strongly compensatory activity that masks the effects of single *elav* mutants [44,45], and only double mutants of mammalian neural Elav factors have been examined to date [19], we suggest that other multiple-knockout conditions may reveal greater collective impacts of ELAV/Hu factors on the neural transcriptome. More generally, our data argue that these classes of 3’ ends can be broadly coregulated and that they may be just two versions of the same process (with splicing playing a comparative minor role in ALE regulation compared to polyadenylation). This may underlie our observation that global ALE-APA and TUTR-APA utilization are broadly correlated in mammals, and may be coregulated by other RBPs [76].

## Materials and methods

### *Drosophila* cell lines and transfections

*Drosophila melanogaster* S2R+ cells were maintained in Schneider’s Insect Medium supplemented with 10% heat inactivated FBS (VWR) and 1% Penicillin-Streptomycin (Thermo Fisher Scientific) at 25°C. Cells were regularly passaged with the density of 2x10^6^/mL. Cell transfection was performed using Effectene (Qiagen) according to the manufacturer’s instructions. Transfections were done using cells at <20 passages with published expression constructs of Flag/HA-tagged wildtype and 3xRRM-Mut versions of Elav, Rbp9 and Fne, and wildtype Sxl, all in pAc5.1C vector [44]. The Uniprot isoforms used were Elav (P16914-1), Rbp9 (Q24474-1), Fne (Q9VYI0-1) and Sxl (P19339-9).

### RNA extraction, reverse transcription PCR and real-time PCR

To extract total RNA from adult *Drosophila* head and body, ~5–10 female or male adult *Drosophila* head or body were pooled together and homogenized in 200 μL TRIzol using a Dounce tissue homogenizer (Thermo Fisher Scientific); To extract total RNA from 1^st^ instar larvae, ~30–40 whole 1^st^ instar larvae or pools of dissected CNS from each genotype were homogenized in 200 μL TRIzol using a Dounce tissue homogenizer (Thermo Fisher Scientific). L1 CNS dissections were performed in batches, with temporary storage on ice for <30 min before addition of TRIzol, and then storage at -80°C; To extract total RNA from transfected S2R+ cells, 72 hrs. post transfection, ~1X10^7^ cells were harvested in TRIzol after pelleting and wash. TRIzol was used to extract RNAs from tissue samples and transfected S2R+ cells; TRIzol LS was used to extract RNAs from cell fractionation samples.

Total RNAs were treated with TurboDNase (Invitrogen) prior to reverse transcription. For reverse transcription using RNAs from adult fly tissues and S2R+ cells: 1 μg total RNA was used as input with a two-step reverse transcription using SuperScript III reverse transcriptase (Invitrogen) with either oligo(dT)_18_ priming or random priming, then 1/10 or 1/20 of RT product was used in a single PCR or real-time PCR reaction, respectively. For reverse transcription using RNAs from whole 1st instar larvae, 300 ng total RNA was used as input with a two-step reverse transcription with oligo(dT)_18_ priming, then 1/10 of RT product was used in a single PCR or real-time PCR reaction. To quantify ALE genes using total RNA from S2R+ cells, raw Ct values were normalized to *rpl32*. To quantify ALE genes using chromatin-associated RNA from S2R+ cells, raw Ct values were normalized to *roX2*. When quantifying ALE genes using 4sU-labeled RNA extracted from S2R+ cells, raw Ct values were normalized to *roX2*. When quantifying ALE genes using total RNA from whole 1st instar larvae, raw Ct values were normalized to *rpl14*. Primers used for RT-PCR and RT-qPCR analysis are listed in **S5 Table**.

### Cell fractionation and isolation of nascent transcripts

72 hours post-transfection, S2R+ cells were harvested and washed three times with PBS. Cell fractionation was performed as described [77]. Briefly, cells were lysed in hypotonic buffer (15 mM HEPES pH 7.6, 10 mM KCl, 5 mM MgOAC, 3 mM CaCl_2_, 300 mM sucrose, 0.1% Triton X-100, 1 mM DTT, 1X complete protease inhibitors) to rupture cell membranes and release nuclei. Nuclei were purified by centrifugation through sucrose cushion to remove intact cells, cell debris, etc., then further lysed with nuclear lysis buffer (10mM HEPES-KOH, pH 7.6, 100 mM KCl, 0.1 mM EDTA, 10% glycerol, 0.15 mM Spermine, 0.5 mM Spermidine, 0.1 M NaF, 0.1 M Na_3_VO_4_, 0.1 mM ZnCl_2_, 1 mM DTT, 1X complete protease inhibitors, 1 U/μL SuperaseIn). Then, 2X NUN buffer (25 mM HEPES-KOH pH 7.6, 300 mM NaCl, 1 M Urea, 1% NP-40, 1 mM DTT, 1X complete protease inhibitors, 1 U/μL SuperaseIn) was added to the suspension with 1:1 ratio to nuclear lysis buffer. After centrifugation, the supernatant comprises nuclear lysate while the pellet contains DNA/histones/Pol II-RNA containing nascent RNA transcripts. We saved 5% of each fraction for western blot analysis, and subjected the remainder to RNA extraction using TRIzol LS (Invitrogen).

### 4sU-labeling and 4sU-containing transcripts isolation

Cells were cultured in medium supplemented with 100 μM of 4sU for 1 hr. before harvest. Total RNA was extracted using TRIzol. 4sU-labeled and pre-existing RNA populations were separated as described [78]. Briefly, 100 μg of total RNA was diluted in 1X Biotinylation buffer (100 mM Tris pH 7.4, 10 mM EDTA) with biotin-HPDP (1 μg/μl in DMF), incubated at room temperature on a rotator for 1.5 hrs. RNA was then extracted with Phenol: Chloroform: Isoamyl Alcohol and precipitated in EtOH for at least 2 hrs.; RNA pellet was dissolved in 50 μl of nuclease-free H_2_O and denatured by incubation at 70°C for 2 min. After chilling on ice, RNA was mixed with 50 μl of pre-washed Streptavidin C1 Dynabeads in 2X bind and wash buffer (10 mM Tris-HCl pH 7.5, 1 mM EDTA, 2 M NaCl, 1 U/μl SuperaseIn). The mixture was incubated on a rotator at room temperature for 1 hr. After incubation, beads were collected on a magnetic stand, and supernatant containing the pre-existing RNAs was discarded. The beads were washed three times with 0.5 ml 1x bind and wash buffer (5 mM Tris-HCl pH 7.5, 0.5 mM EDTA, 1 M NaCl) at 65°C, followed by three washes with 1X bind and wash buffer at room temperature. After complete removal of the bind and wash buffer, beads were resuspended in 200 μl of 1x bind and wash buffer containing 100 mM DTT, and incubated at room temperature for 3 min to elute 4sU-labeled RNA. The elution process was repeated and the eluted RNA was combined and precipitated as described above. The isolated RNA was used for RT-qPCR.

### Identification and quantitation of alternative splicing events from RNA-seq

Total RNA-seq libraries were prepared from S2 cells transfected with actin-based ELAV/Hu family constructs (Flag-HA tagged-Elav/Fne/Rbp9) or mutant counterparts bearing point mutations in all three RRM domains of each factor (3xMut). We used the TruSeq Stranded Total RNA Library Preparation Kit (Illumina) with 2 μg input RNA as starting materials. Final cDNA libraries were sequenced on Illumina HiSeq-1000 sequencer with PE-100 mode. RNA-seq libraries of wildtype, *elav[5]* and *elav[5]/fne[Δ]* mutant L1-CNS were previously described [44].

To identify and quantify alternative splicing events, we used rMATS (v 3.2.5) [53] with -novelSS 1 parameter to detect unannotated splice sites. Among the loci with more than 5 junction reads, FDR<0.05 and ΔPSI>0.3 junction-spanning read counts for each alternative splicing event, including alternative 5′ splice site (A5SS), skipped exon (SE), mutually exclusive exons (MXE), retained intron (RI), and alternative 3’ splice site (A3SS) were identified. We only considered the skipped exon events, and the ΔPSI of each exon was obtained as the difference between the average PSI from the replicates. Elav, Fne, and Rbp9 regulated exons in S2 cells used for downstream analysis were obtained as a union of FDR < 0.05, ΔPSI > 0.3 cassette exons from the comparison between each WT and corresponding mutant variant.

### Identification and quantitation of alternative last exon events from 3’-end sequencing

3’-end sequencing libraries of both S2 and L1-CNS samples were previously described [44]. We mapped the sequenced reads to the *Drosophila melanogaster dm6* reference assembly and resulting reads were clustered and quantified within a 25 bp window as described [60].

To quantify alternative last exon usage by 3’-end cluster reads, we first identified genomic coordination of a specific 3’ UTRs generated by alternative last exon events (Unique 3’UTR). The unique 3’ UTRs which overlap the intronic regions of another isoform were identified, and the longest 3’ UTR was selected among the isoforms with the same 3’UTR start sites. Sum of the 3’-end cluster counts from the longest unique 3’ UTR was used for ALE events quantification.

To calculate ALE usage ratio, we first selected the unique 3’ UTR that is used dominantly in the control, and calculated the utilization rates of the proximal unique 3’ UTR and distal unique 3’ UTR in both control and sample based on the dominant universal unique 3’ UTR. ALE usage represents the value obtained by dividing the sum of universal unique 3’ UTR and distal unique 3’ UTR by the sum of universal unique 3’ UTR and proximal unique 3’ UTR, and the ALE usage ratio is the divided value of the ALE usage of sample and control. Statistical tests were done by two-way ANOVA, and *p* <0.05 and ratio difference 2-fold was used for significance cutoff.

### Relative strengths of internal ALE terminal ends

We calculate the bypass score for each ALE 3’-ends (internal ALE terminal ends) by percentage of sum of 3’-seq reads on the unique 3’ UTR downstream of a given 3’ end. The bypass ratio was calculated by comparing the bypass score of each ALE 3’-end between two samples in order to compare relative strength of ALE 3’-end. Bypass ratio 1 means that the ALE 3’-end has as much downstream last exons compared to the control and a ratio 0 means that the total amount of downstream last exon is not changed in both samples. Conversely, the unbypassed ratio is a measure of how much ALE 3’-end of a sample is not bypassed compared to the control.

### BrdU-CLIP sequencing library preparation

BrdU-CLIP libraries were made using S2R+ cells with overexpression of wildtype Elav or Elav-3xRRM-mut based on a published protocol [79] with some optimizations. Protein-RNA complexes were immunoprecipitated using anti-FLAG antibody (Sigma Aldrich) followed by stringent washes and ligation of the 3’ linker. After digestion of protein, the RNA was purified and reverse transcribed using a dNTP mix where dTTP is replaced with BrdUTP. The RT primer has 5’ and 3’ anchor sequences used later for PCR amplification separated by an APE1 cleavage site. The BrdUTP incorporated into the resulting cDNA allows on-bead cDNA purification using anti-BrdU antibody (Sigma Aldrich). The purified cDNA is then circularized using CircLigase II and real-time PCR performed using SYBR green staining. The PCR reaction is stopped when the fluorescence intensity (RFU) reaches ~250-500. Single wildtype and 3xRRM Elav-CLIP libraries were sequenced by Illumina Hi-Seq 2000 to obtain 51 nt reads.

### BrdU-CLIP data analysis

Raw CLIP-seq reads were processed by trimming of adaptors by Cutadapt v1.18, and all reads were required to have a length of at least 29 bp containing 14 nt random barcode. The filtered reads were collapsed then random barcodes were trimmed. Remaining reads were mapped to unique positions in the *Drosophila melanogaster* genome (UCSC version dm6) using Burrows-Wheeler Aligner (BWA 0.7.17), allowing mismatches depending on read length with parameter -n 0.06. Peaks were subsequently called from uniquely mapping reads by CLIP Tool Kit (CTK) v1.1.3 with default parameters [80]. The peak summit was used as the reference binding site and peaks were ranked based on the number of reads at the summit. Meta-gene analysis was performed using deeptools v3.3.0 [81] to search the CLIP peak distribution of indicated genomic locus.

### Motif analysis

We used MEME-suite (v 5.0.2) to discover *de novo* motifs arounds Flag-Elav BrdU-CLIP peaks and 50-nucleotide windows downstream of the cleavage site and 100-nucleotide windows of each up and downstream of the splicing site with default parameters with 3-order Markov model [82]. For differential motif enrichment, *-objfun* de function was used for discovering motifs in the indicated locus from those control sequences. Local enrichment of position weight matrix (PWM) of given motifs was analyzed by seqPattern R package in Bioconductor (https://bioconductor.org/packages/seqPattern/).

### Analysis of human differentiation time course samples

RNAseq data for human iPSC to mature neurons was downloaded from. https://www.ncbi.nlm.nih.gov/bioproject/?term=PRJNA596331.) [64]. Each sample is derived from one of several different donors and differentiated through multiple timepoints. To quantify 3’ end usage across neuronal differentiation, the LABRAT software package was used.

We used LABRAT [76] to assess alternative 3’ UTR usage in mammalian RNA-seq data. LABRAT quantifies APA events by comparing the expression of the final two exons of every expressed transcript and calculating a *ψ* value that relates relative APA site usage (https://github.com/TaliaferroLab/LABRAT/). To simplify LABRAT output, only gene models with two APA isoforms were considered. For these genes, *ψ* values of 0 indicate exclusive usage of the proximal APA site while *ψ* values of 1 indicate exclusive usage of the downstream APA site.

To define tandem UTR and ALE gene models, LABRAT observes the isoform structures at the 3’ end of a gene. If all APA sites are contained within the same exon, then the structure in tandem UTR. If all APA sites are contained within different exons, then the structure is ALE. If a gene has more than two APA sites, it is possible for the gene to fit into neither classification. In these cases, LABRAT assigns the gene to have a “mixed” structure. These genes were not considered in following analyses.

To define genes that change their APA usage through neuronal differentiation by using more downstream APA sites, we utilized standard k-means clustering. Genes with changing 3’ end usage across timepoints were clustered by their mean *ψ* value revealing a distinct cluster of genes with increasing *ψ*. These genes were termed as having “neuronally restricted” (NR) long isoforms. These include 706 genes with shifts towards longer 3’ UTRs within terminal 3’ UTRs (TUTR), 269 genes with shifts towards distal alternative last exons (ALE), and 15 genes with mixed TUTR/ALE profiles; the small latter class was not considered further in our analysis. A control group of genes was defined as being expressed in the later differentiation time points (Day 47, 63, and 77) but either had no change in *ψ* or showed decreasing *ψ* values across the differentiation time course.

Sequences flanking the proximal PAS (100 nucleotides upstream and downstream) were analyzed for each NR and control gene. Uridine content was plotted and compared using Wilcoxon rank-sum tests and considered significant with a p < 0.05. Additionally, these sequences were analyzed for ELAVL PWM motif matches of greater than 80%. ELAVL motifs were obtained from cisbpRNA database [58] and RNA bind-n-seq results [83]. Multiple motifs represent each of the 4 protein family members and motifs can be quite similar. Motifs with sufficient match to a sequence were centered onto a single nucleotide. If a similar motif matched at the exact location, it was not counted twice. Every motif count for an ELAVL protein was summed across nucleotide positions for the NR and control gene groups. Motif frequency within the groups was plotted and compared using Wilcoxon rank-sum tests and considered significant with a p < 0.05.

## Supporting information

S1 Fig Analysis of changes in alternative splicing events following manipulation of *Drosophila* ELAV/Hu RBPs. (A-B) The stacked bar plots categorize the numbers of all differential alternative splicing events involving internal exons. Note that exon skipping events are the most numerous category of alternative splicing event affected by Elav/Hu RBP manipulation. (A) Gain-of-function of ELAV/Hu RBPs in S2 cells. The effects of each WT ELAV/Hu RBP (Elav/Fne/Rbp9) were compared to their RNA binding defective counterparts (3X-Mut). (B) Loss of function of ELAV/Hu RBPs in 1st instar larval CNS (L1-CNS). Pairwise comparisons between wildtype *Canton-S*, single *elav[5]* null and double *elav[5]/Δfne* null mutants. (C) Distribution of ΔPSI values of the WT-GOF induced excluded (*left*) and included (*right*) exons were compared to the S2.
(TIF)

S2 Fig Examples of exon skipping and exon inclusion promoted by Drosophila ELAV/Hu RBPs. (*Top*) Three examples of genetic sufficiency and necessity for exon skipping promoted by ELAV/Hu RBPs. GOF of wildtype Elav/Fne/Rbp9 (but not their RNA-binding defective counterparts) promotes skipping of an alternative cassette exon in S2 cells, while combined LOF of elav/fne results in inclusion of the same exon in L1-CNS. (*Bottom*) Three examples of genetic sufficiency and necessity for exon inclusion promoted by ELAV/Hu RBPs. GOF of wild- type Elav/Fne/Rbp9 (but not their RNA-binding defective counterparts) promotes inclusion of an alternative cassette exon in S2 cells, while combined LOF of elav/fne results in exclusion of the same exon in L1-CNS.
(TIF)

S3 Fig Examples of *elav/fne*-regulated alternatively spliced cassette exons. This figure compares exons with deregulated usage in *elav/fne* mutant, annotated only in Carrasco and Hilgers (Mol Cell 2020) or only in this study. IGV data tracks include RNA-seq data from control and *elav/fne* embryos and head Elav-CLIP data (from Carrasco and Hilgers) and RNA-seq data from control and *elav/fne* embryos and CLIP data of wt and RRM-mut Elav expressed in S2 cells (this study). (A) Numbers of *elav/fne*-regulated excluded (*left*) and included (*right*) exons annotated in this study or by Carrasco and Hilgers. (B) Representative *elav/fne*-regulated exons annotated only by Carrasco and Hilgers show little changes in RNA-seq data, but have local flanking Elav CLIP. (C-D) Representative exon-exclusion (C) and exon-inclusion (D) events in *elav/fne* mutants annotated only in this study, but not by Carrasco and Hilgers. Some, but not all of these have flanking intronic Elav CLIP in S2 cell data; but most have broad Elav-CLIP patterns from heads.
(TIF)

S4 Fig Subcellular localization of ectopic ELAV/Hu RBPs. (A) Scheme for ectopic expression of wt and RRM mutant ELAV/Hu RBPs, and other U-rich RBPs. These cell materials were subjected to the fractionation as shown to yield cytoplasmic (C) and nuclear (N) fractions analyzed here, as well as chromatin fractions (Chr) analyzed in Fig 5. (B) Western blotting analysis of fractionated, transfected cells. We use HP1 to mark nuclear fractions and ß-tubulin to mark cytoplasmic fractions, and use the HA tag to follow the distribution of the RBPs (or control GFP). Note that an apparent non-specific band is labeled by HP1 antibody (marked by pink asterisks, *), based on the inconsistent size and aberrant distribution. As seen, HP1 is largely segregated to nuclear fractions while ß-tubulin is found in cytoplasmic fractions. These analyses show that ectopically expressed ELAV/Hu RBPs (Elav, Elav-RRM3xmut, Rbp9 and Fne) can all be detected in cytoplasm and nuclei. While these do not mirror the reported endogenous distributions in neurons, we also know that endogenous Elav can be detected in cytoplasm and Fne is substantially relocalized into nuclei in *elav* mutant neurons. Note also that ectopic ELAV/Hu proteins seem to produce truncated products in the cytoplasm, based on their smaller sizes than full-length ELAV/Hu RBPs (marked by aqua asterisks, *). In any case, these data provide a molecular basis that ectopic expression of actin>Elav/Fne/Rbp9 constructs are capable of inducing similar splicing and ALE changes in S2 cells.
(TIF)

S5 Fig Specificity of ELAV/Hu RBPs to induce alternative splicing. *LIMK1* exhibits tissue specific alternative splicing of a cassette exon, as assayed by rt-PCR using primers for its flanking exons. (*Left*) *LIMK1* is subject to inclusion in heads (H) but not bodies (B); note these data are shown in main Fig 3B and are included here for reference. (*Right*) S2 cells were transiently transfected with wild-type ELAV/Hu RBPs (Elav, Rbp9 and Fne), their 3x-RRM mutant counterparts (mut), other U-rich binding RBPs (Sxl, Ssx, hnRNPC) and CstF64; GFP and empty vectors were used as additional negative controls. Only wt-ELAV/Hu RBPs induced alternative splicing of *LIMK1*, causing usage of the neural splice isoform in S2 isoform in S2 cells.
(TIF)

S6 Fig Flanking intronic features associated with exon exclusion and skipping regulated by Drosophila ELAV/Hu RBPs. (A) Shown are nucleotide frequencies and de novo motif searches from flanking intronic regions of regulated cassette exons from ELAV/Hu RBP GOF in S2 cells (*top*) or elav/fne LOF in L1-CNS (*bottom*). These are separated according to exons that are either included or excluded in the corresponding manipulated condition, bearing in mind that exons that are included in elav/fne LOF correspond to ones that are excluded in the wildtype condition (since these factors are normally expressed in CNS). We observe overt enrichment of upstream intronic uridine (blue oval) amongst introns that are excluded by misexpression of Elav/Fne/Rbp9 (EFR) in S2 cells or are included in elav or elav/fne knockout L1-CNS. This is associated with strong enrichment of ELAV/Hu-type binding sites from de novo motif searches in their upstream flanking introns, as well as a lesser but still significant enrichment of such sites in flanking downstream intronic regions. Exons that are included by ELAV/Hu RBP activity do not show such flanking uridine enrichment or ELAV/Hu-type binding sites, but instead show enrichment of downstream flanking adenosine (red oval). (B) Result of differential motif enrichment analysis from 100 nt flanking intronic regions of Elav/Hu RBP GOF induced excluded exons compared to included exons in S2 cells (*left*), and elav/fne LOF induced included exons compared to excluded exons (*right*).
(TIF)

S7 Fig Additional examples of distal alternative last exon splicing usage promoted by *Drosophila* ELAV/Hu RBPs. These four genes generally share the features of the distal ALE isoforms that are (1) preferentially expressed in head compared to other tissues (detection in carcass may correspond to presence of ventral nerve cord in these dissected samples), (2) are developmentally induced during the timecourse of embryogenesis, (3) are induced in S2 cells upon transfection of wildtype Elav/Rbp9/Fne but not their RRM-mutant (Mut) counterparts, and (4) are expressed in dissected wildtype *Canton-S* and *elav[5]* null L1-CNS, but not in *elav[5]/Δfne* double mutant L1-CNS. Tracks are labeled as to 3’-seq or RNA-seq data.
(TIF)

S8 Fig Examples of *elav/fne*-regulated APA and ALE genes. This Figure compares RNA-seq and 3’-seq patterns of APA or ALE genes annotated in this study and Carrasco and Hilgers (Mol Cell 2020). These examples highlight the combined utilities of appropriate developmental stage, isolated nervous system, 3’-seq evidence and reciprocal loss- and gain-of-function data, to annotate alterations of 3’ isoforms with high confidence. 3’-UTRs are highlighted by yellow shade. (A) elav/fne-regulated APA (*left*) and ALE (*right*) genes identified by in this study and Carrasco and Hilgers (Mol Cell 2020). (B) UTR-APA targets in Carrasco and Hilgers (Mol Cell 2020), not in this study. rad is an example of a gene altered only in the early embryo data, but not late embryo data. It was not much changed in our CNS or S2 datasets. Vha26 is not substantially altered in any datasets. (C) ALE-APA (= CDS-APA) targets in Carrasco and Hilgers (Mol Cell 2020), not in this study. Almost none of these annotations exhibit substantial ALE usage changes in any datasets. (D) Example loci with deregulation of terminal 3’ UTR APA isoforms in *elav/fne*-mutants, annotated only in this study. (E) Example loci with deregulation of 3’ UTR ALE isoforms in *elav/fne*-mutants, annotated only in this study.
(TIF)

S9 Fig Validation of terminal 3’ UTR extensions respond to ELAV/Hu RBPs. S2 cells were transfected with wildtype (WT) or mutant (Mut) versions of Elav/Rbp9/Fne; the latter constructs contain point mutations in all three RRM domains. qPCR of total RNA samples for universal (uni) and extension (ext) 3’ UTR amplicons shows that the 3’ UTRs of *tai* and *ctp* are specifically extended by ectopic wt ELAV/Hu RBPs. Measurements were normalized to *rpl32*.
(TIF)

S10 Fig Analysis of polyadenylation signals (PAS) within ALE genes. (A) Schematic of ALE gene models and designation of PAS locations. (B) PAS types in S2-expressed genes with a single 3’ end. A strong majority of genes utilize the canonical AAUAAA PAS or one of its top two *Drosophila* variants (AUUAAA/AAUAUA). (C) In S2 cells, ALE-expressed genes exhibit lower frequency of canonical PAS at their proximal ALE 3’ ends than the single-end gene reference. However, these are not substantially different between loci that are bypassed in the presence of ectopic ELAV/Hu RBPs and ones that are not. (D) In L1-CNS, ALE-expressed genes exhibit lower frequency of canonical PAS at their proximal ALE 3’ ends than the single-end gene reference. However, these are not substantially different between loci that are bypassed in the presence of ectopic ELAV/Hu RBPs and ones that are not. Note that in both cell/tissue cohorts, "other PAS" exhibit overall lower quality features, which is consistent with these collectively including a portion of biochemically valid although potentially less biologically critical PAS.
(TIF)

S11 Fig ELAV/Hu binding sites are highly enriched downstream of pA sites of proximal ALE models that are bypassed by ELAV/Hu RBPs. (A) Shown are results of de novo motif analysis from 50 nt regions downstream of various cohorts of polyadenylation (pA) sites from ALE gene models of loci expressed in S2 cells or L1-CNS. Significant motifs recovered at >10% frequency are shown. (*Top*) In S2 cells, proximal ALEs that were prone to being switched to distal ALEs in the presence of wt Elav/Fne/Rbp9 are highly enriched for downstream ELAV/Hu-type binding sites. Note that there is also enrichment for similar sites downstream of unchanged proximal ALE pA sites, albeit at a much lower frequency, potentially indicating that ELAV/Hu RBPs may be involved in their regulation but were insufficient to mediate their switching in these experimental tests. (*Bottom*) In L1-CNS, the only motif recovered downstream of proximal ALE PAS that became aberrantly switched from distal ALE usage in *elav/fne* double mutants was the ELAV/Hu-type site. Similar to the S2 tests, we also observed significant, but much less frequent, enrichment of similar sites downstream of proximal ALE PAS that were not affected in *elav/fne* mutants. Again, this may indicate that the involvement of ELAV/Hu RBPs is broader than functionally detected in these data, either because of the need for the triple mutant or because of other parallel regulatory mechanisms. (B) Result of differential motif enrichment analysis from 50 nt regions downstream of Elav/Hu-regulated ALE pA sites compared to unregulated ALE pA sites. U-rich motifs are relatively enriched to downstream of regulated ALE pA sites for both S2 (*left*) and L1-CNS (*right*).
(TIF)

S12 Fig Distribution of wt and mutant Elav CLIP reads and peaks in S2 cells. BrdU CLIP-seq data was generated from S2 cells expressing WT and 3xRRM-mut (Mut) Elav, conditions that generate characteristic redeployment of splicing and 3’ UTR isoforms (eg. Figs 2–6). These pie charts represent data with rRNA reads filtered out. Left, distribution of reads (also shown in Fig 7A) and right, distribution of peaks. The read distribution reflects a strong bias of Elav-wt CLIP data to 3’ UTRs, as reflected in the metagene profile (see Fig 7B), but when counting peaks (regardless of size), we can see that there is peak coverage elsewhere in gene models. This underlies the ability to analyze RRM-dependent Elav CLIP occupancy in mRNA isoform generation from these data.
(TIF)

S13 Fig De novo motif discovery analysis of the Elav BrdU-CLIP binding sites. (A) Logo representation of the five top-scoring motifs from transcriptome-wide Elav BrdU CLIP clusters. For the wild-type Elav CLIP-seq, a significant U-rich motif was recovered with a high frequency, whereas any U-rich motif was found from its mutant variant. (B) Elav binding motif identified from the peaks arounds 300 nt downstream of proximal pA sites.
(TIF)

S14 Fig Psi value changes for genes with alternative 3’ ends across human iPSC-neuronal differentiation. Each row of a timecourse plot shows the distribution of LABRAT calculated psi values across loci relative to the starting day 02 dataset. The top plots of genes with neural-restricted (NR) distal ALE-switching (left) or terminal 3’ UTR (TUTR) lengthening (right) are the same as in Fig 7C and 7D and shown for reference. The bottom plots comprise the control sets of ALE and TUTR gene used for analysis in Fig 8.
(TIF)

S1 Table Catalog of alternatively spliced cassette exons with altered usage in gain- and loss-of-conditions of ELAV/Hu RBPs. (XLSX)

S2 Table Catalog of alternative last exons with altered usage in gain- and loss-of-conditions of ELAV/Hu RBPs. (XLSX)

S3 Table Numbers of bound transcripts in Elav-CLIP data from S2 cells (wt and RRM-mutant Elav, this study) and heads (endogenous Elav, Carrasco and Hilgers, Mol Cell 2020), at different cutoffs. (XLSX)

S4 Table Human genes with neuronal-specific alteration of alternative last exon and terminal 3’ UTR isoforms, as determined from in vitro differentiation of iPSCs into neurons. (XLSX)

S5 Table Oligonucleotides used in this study to analyze regulated mRNA isoforms, genotype mutants, and prepare CLIP libraries. (XLSX)
