# Supplementary figures and images for: ELAV/Hu RNA binding proteins determine multiple programs of neural alternative splicing
Source: PLoS Genet. 2021 Apr 7;17(4):e1009439. doi: 10.1371/journal.pgen.1009439 (PMC8055025; doi:10.1371/journal.pgen.1009439)

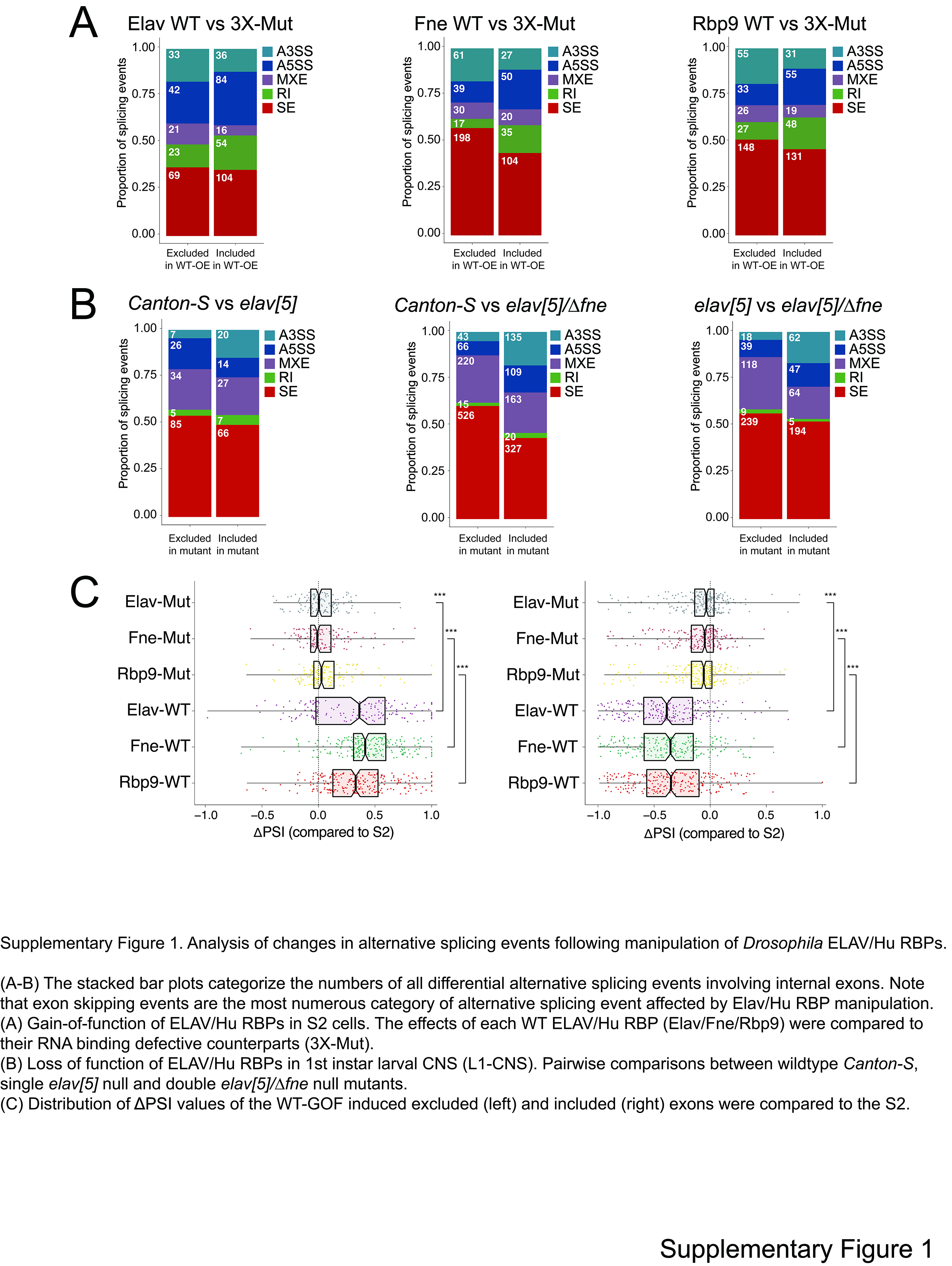

Supplement: S1 Fig — (A-B) The stacked bar plots categorize the numbers of all differential alternative splicing events involving internal exons. Note that exon skipping events are the most numerous category of alternative splicing event affected by Elav/Hu RBP manipulation. (A) Gain-of-function of ELAV/Hu RBPs in S2 cells. The effects of each WT ELAV/Hu RBP (Elav/Fne/Rbp9) were compared to their RNA binding defective counterparts (3X-Mut). (B) Loss of function of ELAV/Hu RBPs in 1st instar larval CNS (L1-CNS). Pairwise comparisons between wildtype Canton-S, single elav[5] null and double elav[5]/Δfne null mutants. (C) Distribution of ΔPSI values of the WT-GOF induced excluded (left) and included (right) exons were compared to the S2. (TIF) [file pgen.1009439.s001.tif]

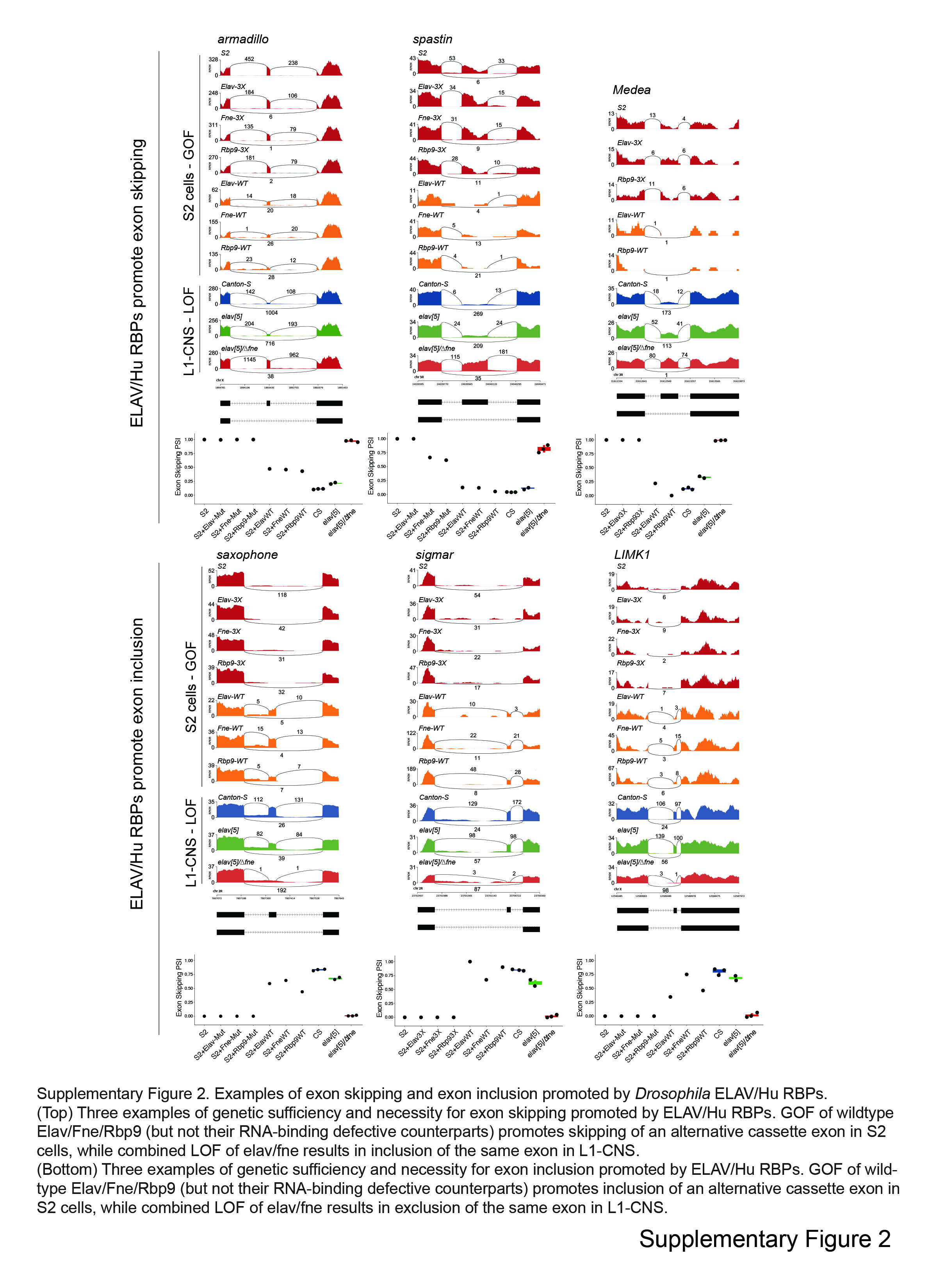

Supplement: S2 Fig — (Top) Three examples of genetic sufficiency and necessity for exon skipping promoted by ELAV/Hu RBPs. GOF of wildtype Elav/Fne/Rbp9 (but not their RNA-binding defective counterparts) promotes skipping of an alternative cassette exon in S2 cells, while combined LOF of elav/fne results in inclusion of the same exon in L1-CNS. (Bottom) Three examples of genetic sufficiency and necessity for exon inclusion promoted by ELAV/Hu RBPs. GOF of wild- type Elav/Fne/Rbp9 (but not their RNA-binding defective counterparts) promotes inclusion of an alternative cassette exon in S2 cells, while combined LOF of elav/fne results in exclusion of the same exon in L1-CNS. (TIF) [file pgen.1009439.s002.tif]

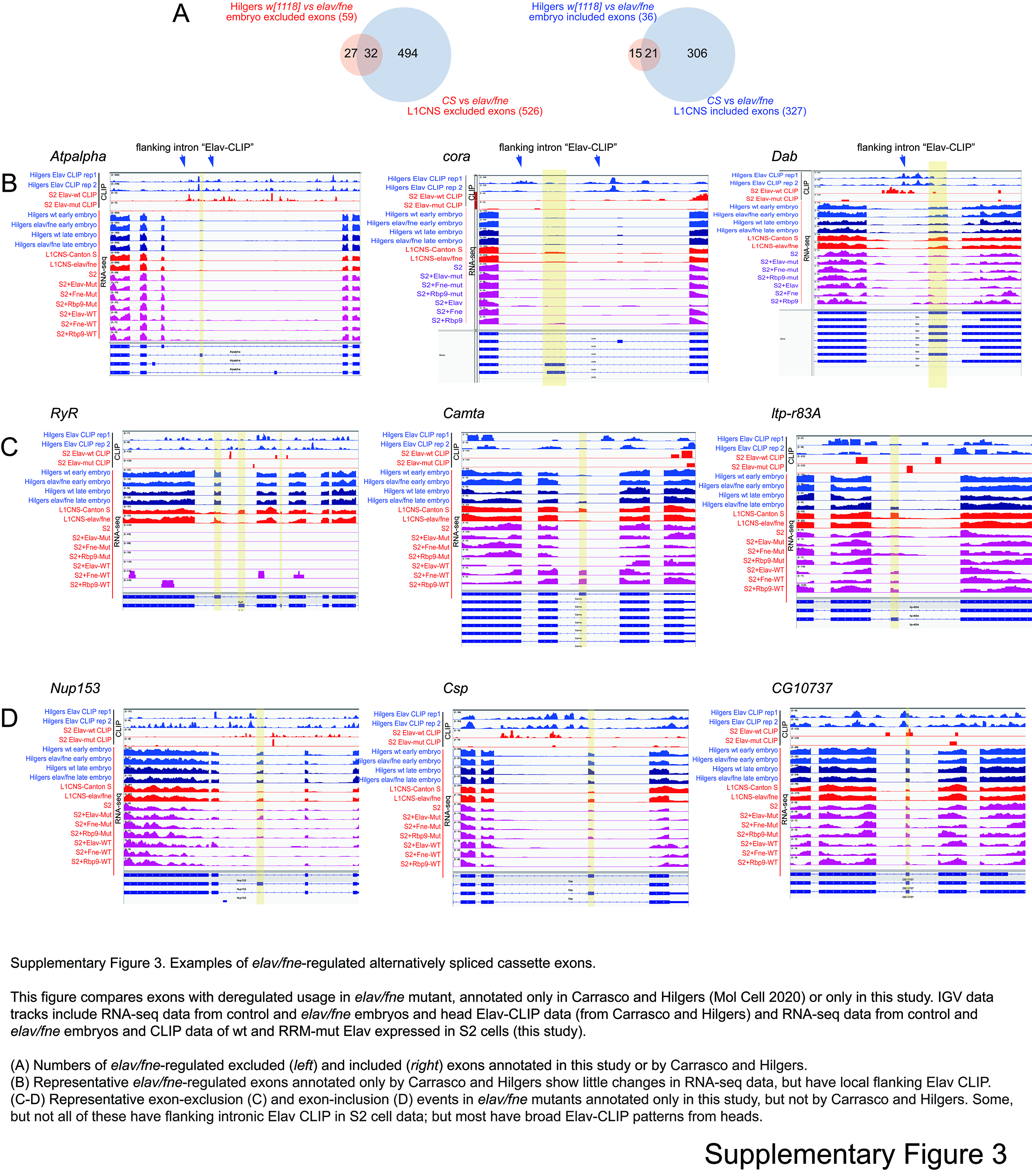

Supplement: S3 Fig — This figure compares exons with deregulated usage in elav/fne mutant, annotated only in Carrasco and Hilgers (Mol Cell 2020) or only in this study. IGV data tracks include RNA-seq data from control and elav/fne embryos and head Elav-CLIP data (from Carrasco and Hilgers) and RNA-seq data from control and elav/fne embryos and CLIP data of wt and RRM-mut Elav expressed in S2 cells (this study). (A) Numbers of elav/fne-regulated excluded (left) and included (right) exons annotated in this study or by Carrasco and Hilgers. (B) Representative elav/fne-regulated exons annotated only by Carrasco and Hilgers show little changes in RNA-seq data, but have local flanking Elav CLIP. (C-D) Representative exon-exclusion (C) and exon-inclusion (D) events in elav/fne mutants annotated only in this study, but not by Carrasco and Hilgers. Some, but not all of these have flanking intronic Elav CLIP in S2 cell data; but most have broad Elav-CLIP patterns from heads. (TIF) [file pgen.1009439.s003.tif]

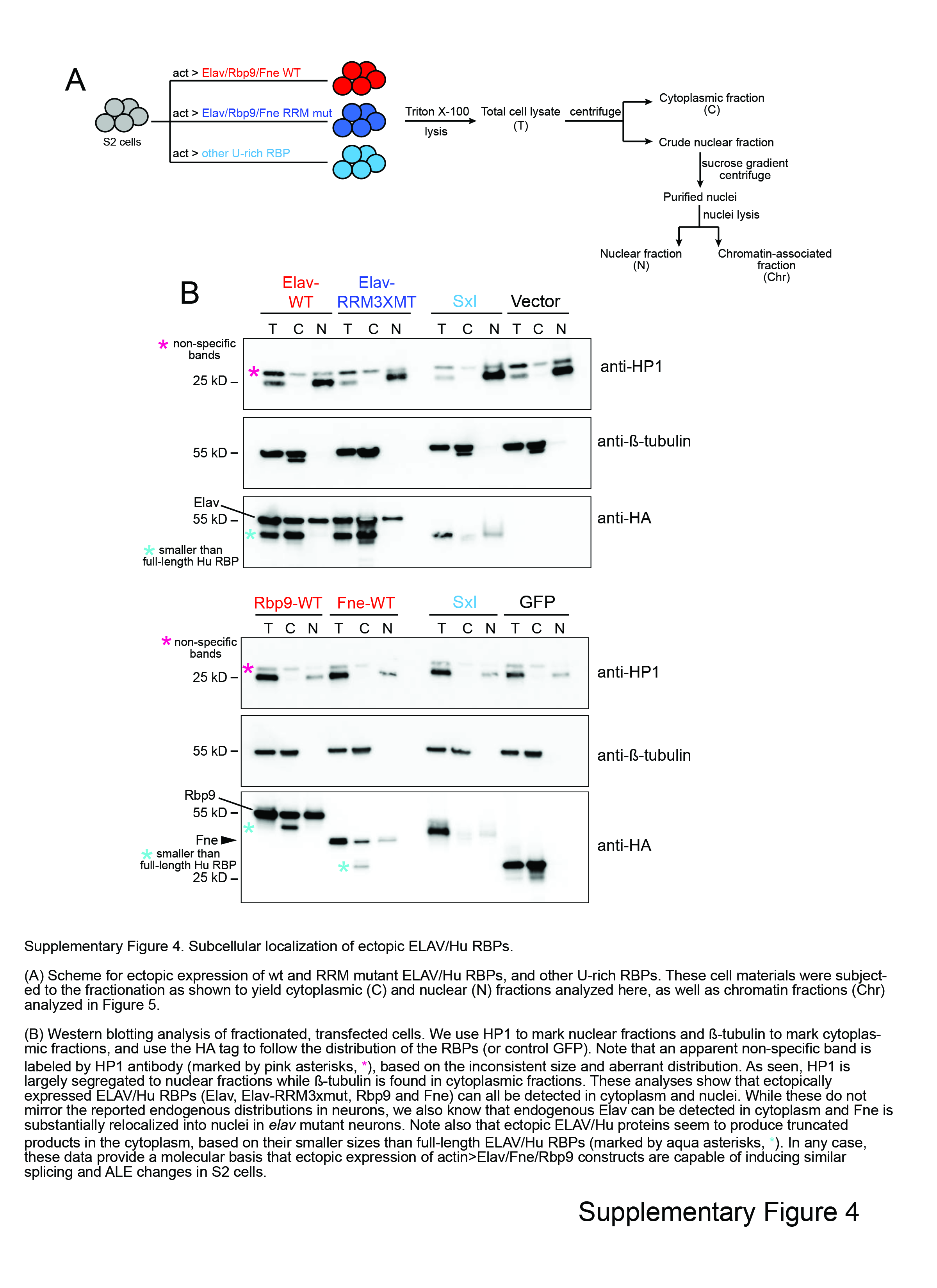

Supplement: S4 Fig — (A) Scheme for ectopic expression of wt and RRM mutant ELAV/Hu RBPs, and other U-rich RBPs. These cell materials were subjected to the fractionation as shown to yield cytoplasmic (C) and nuclear (N) fractions analyzed here, as well as chromatin fractions (Chr) analyzed in Fig 5. (B) Western blotting analysis of fractionated, transfected cells. We use HP1 to mark nuclear fractions and ß-tubulin to mark cytoplasmic fractions, and use the HA tag to follow the distribution of the RBPs (or control GFP). Note that an apparent non-specific band is labeled by HP1 antibody (marked by pink asterisks, *), based on the inconsistent size and aberrant distribution. As seen, HP1 is largely segregated to nuclear fractions while ß-tubulin is found in cytoplasmic fractions. These analyses show that ectopically expressed ELAV/Hu RBPs (Elav, Elav-RRM3xmut, Rbp9 and Fne) can all be detected in cytoplasm and nuclei. While these do not mirror the reported endogenous distributions in neurons, we also know that endogenous Elav can be detected in cytoplasm and Fne is substantially relocalized into nuclei in elav mutant neurons. Note also that ectopic ELAV/Hu proteins seem to produce truncated products in the cytoplasm, based on their smaller sizes than full-length ELAV/Hu RBPs (marked by aqua asterisks, *). In any case, these data provide a molecular basis that ectopic expression of actin>Elav/Fne/Rbp9 constructs are capable of inducing similar splicing and ALE changes in S2 cells. (TIF) [file pgen.1009439.s004.tif]

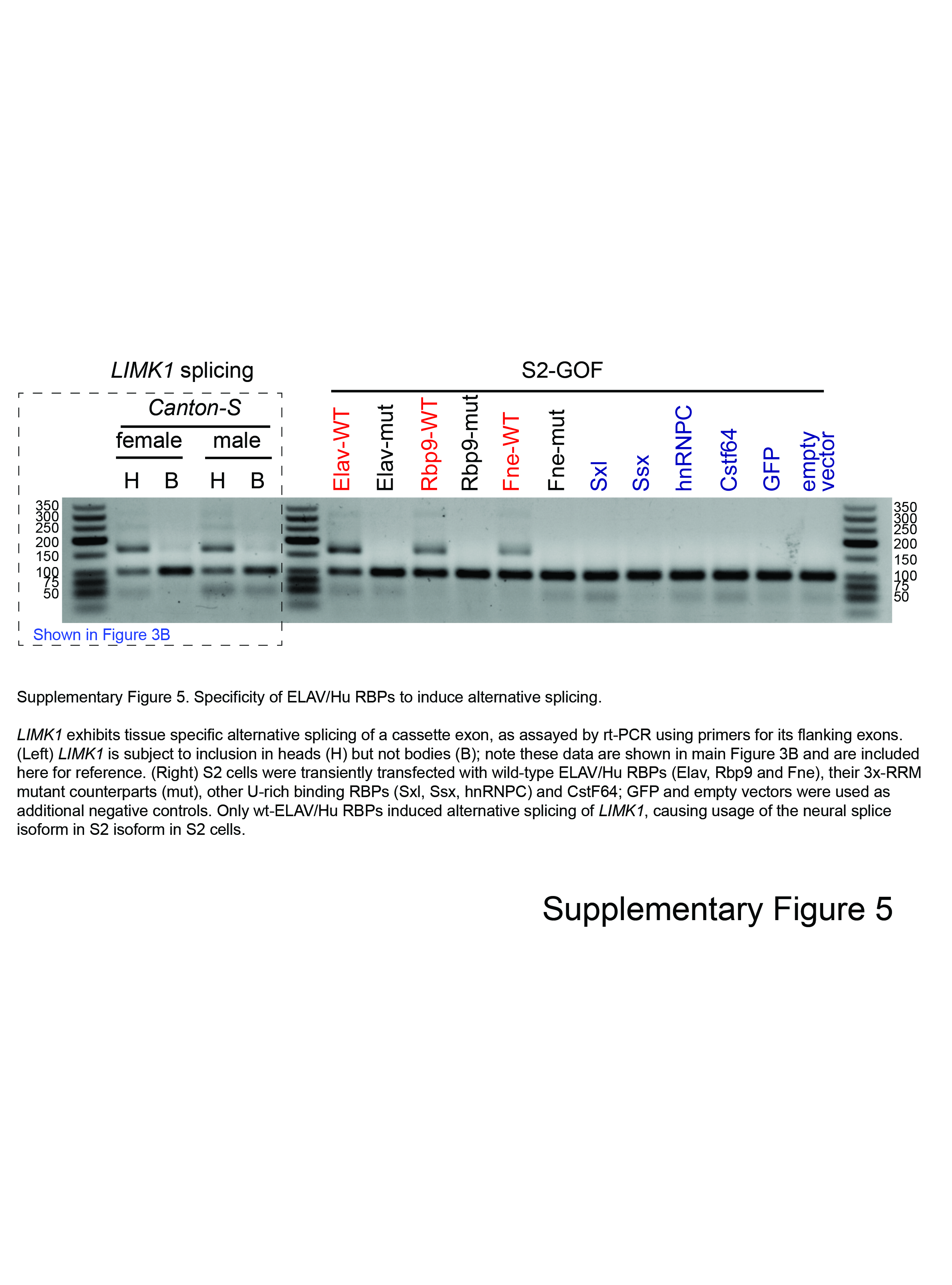

Supplement: S5 Fig — LIMK1 exhibits tissue specific alternative splicing of a cassette exon, as assayed by rt-PCR using primers for its flanking exons. (Left) LIMK1 is subject to inclusion in heads (H) but not bodies (B); note these data are shown in main Fig 3B and are included here for reference. (Right) S2 cells were transiently transfected with wild-type ELAV/Hu RBPs (Elav, Rbp9 and Fne), their 3x-RRM mutant counterparts (mut), other U-rich binding RBPs (Sxl, Ssx, hnRNPC) and CstF64; GFP and empty vectors were used as additional negative controls. Only wt-ELAV/Hu RBPs induced alternative splicing of LIMK1, causing usage of the neural splice isoform in S2 isoform in S2 cells. (TIF) [file pgen.1009439.s005.tif]

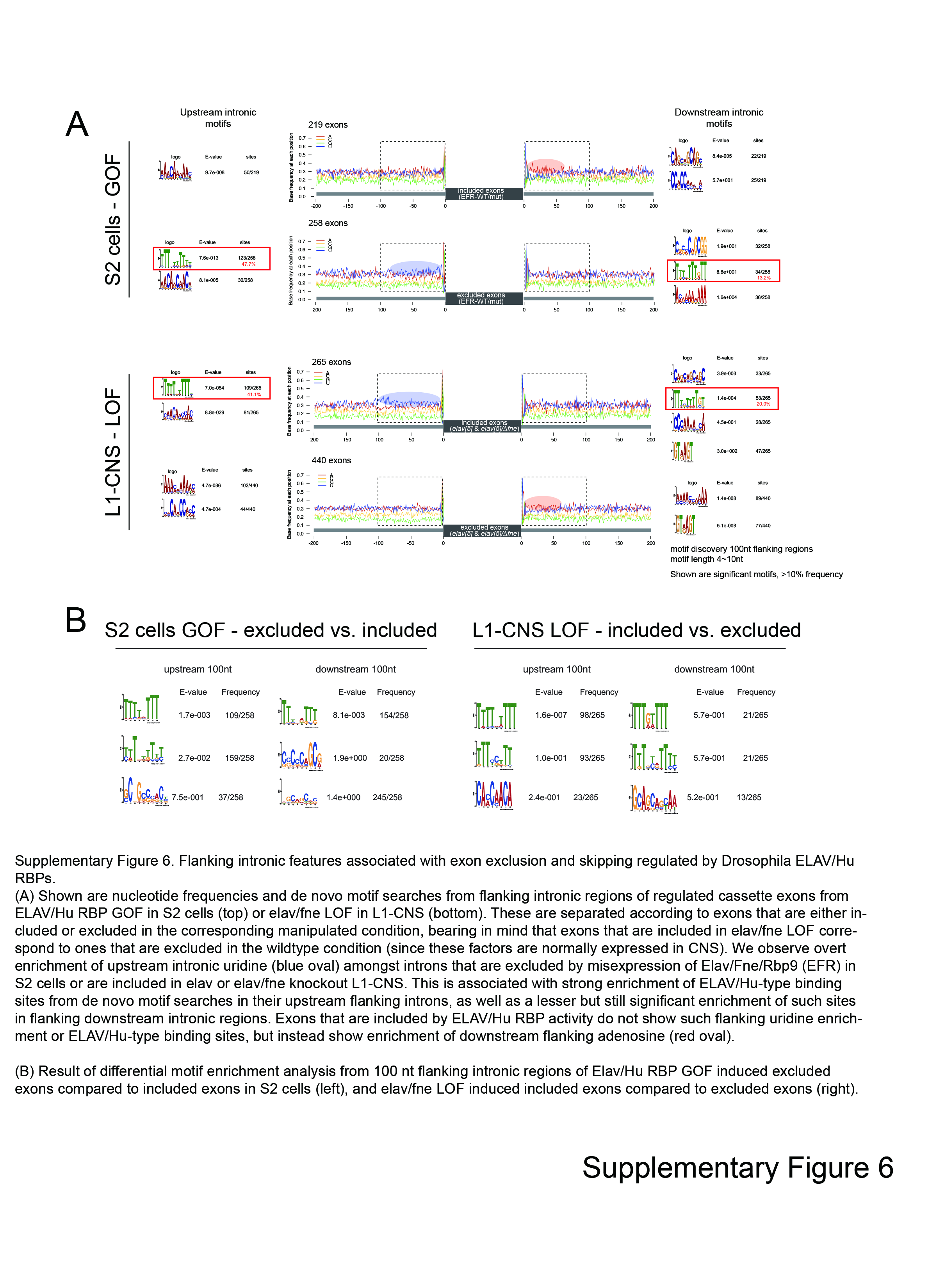

Supplement: S6 Fig — (A) Shown are nucleotide frequencies and de novo motif searches from flanking intronic regions of regulated cassette exons from ELAV/Hu RBP GOF in S2 cells (top) or elav/fne LOF in L1-CNS (bottom). These are separated according to exons that are either included or excluded in the corresponding manipulated condition, bearing in mind that exons that are included in elav/fne LOF correspond to ones that are excluded in the wildtype condition (since these factors are normally expressed in CNS). We observe overt enrichment of upstream intronic uridine (blue oval) amongst introns that are excluded by misexpression of Elav/Fne/Rbp9 (EFR) in S2 cells or are included in elav or elav/fne knockout L1-CNS. This is associated with strong enrichment of ELAV/Hu-type binding sites from de novo motif searches in their upstream flanking introns, as well as a lesser but still significant enrichment of such sites in flanking downstream intronic regions. Exons that are included by ELAV/Hu RBP activity do not show such flanking uridine enrichment or ELAV/Hu-type binding sites, but instead show enrichment of downstream flanking adenosine (red oval). (B) Result of differential motif enrichment analysis from 100 nt flanking intronic regions of Elav/Hu RBP GOF induced excluded exons compared to included exons in S2 cells (left), and elav/fne LOF induced included exons compared to excluded exons (right). (TIF) [file pgen.1009439.s006.tif]

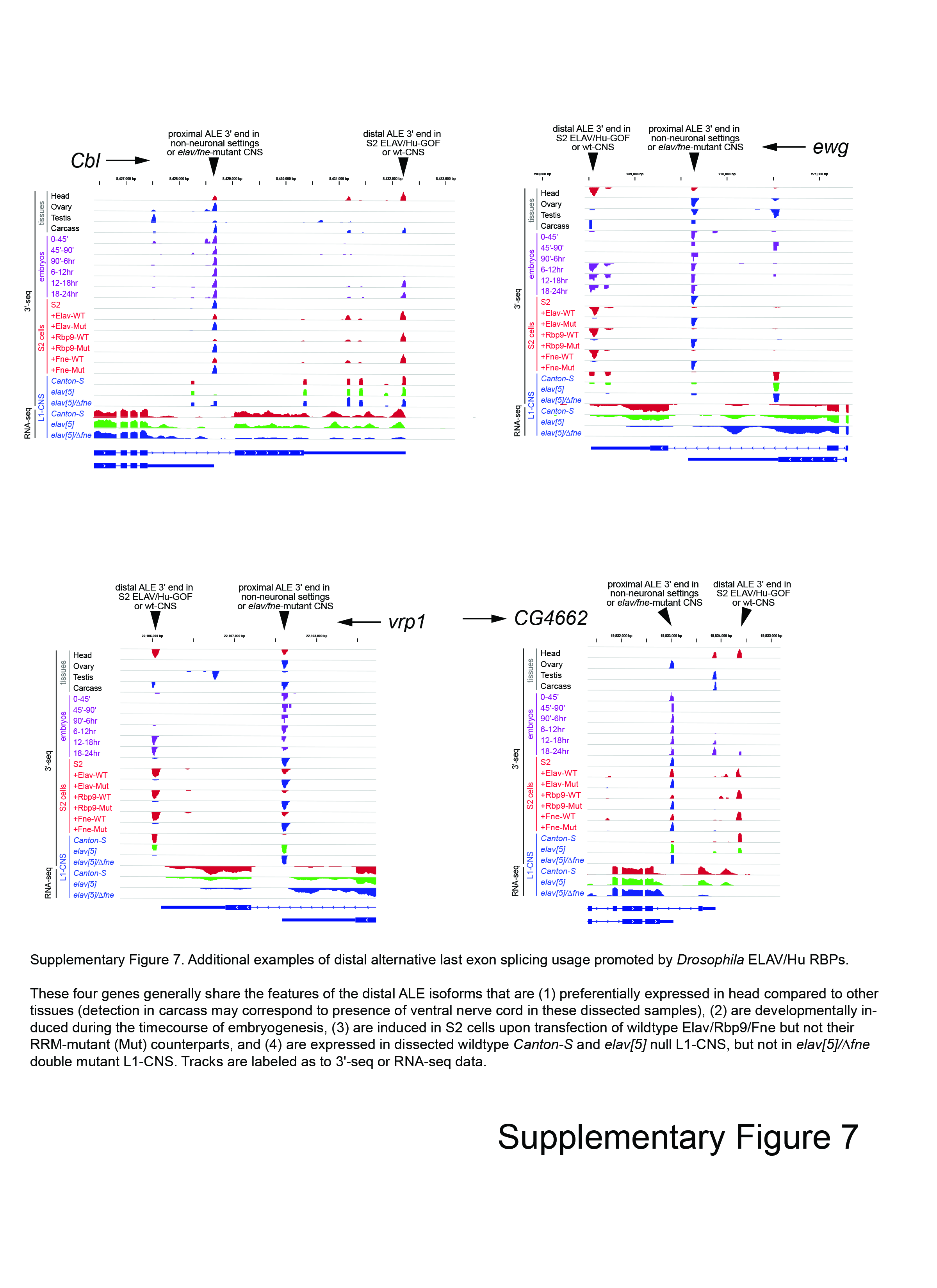

Supplement: S7 Fig — These four genes generally share the features of the distal ALE isoforms that are (1) preferentially expressed in head compared to other tissues (detection in carcass may correspond to presence of ventral nerve cord in these dissected samples), (2) are developmentally induced during the timecourse of embryogenesis, (3) are induced in S2 cells upon transfection of wildtype Elav/Rbp9/Fne but not their RRM-mutant (Mut) counterparts, and (4) are expressed in dissected wildtype Canton-S and elav[5] null L1-CNS, but not in elav[5]/Δfne double mutant L1-CNS. Tracks are labeled as to 3’-seq or RNA-seq data. (TIF) [file pgen.1009439.s007.tif]

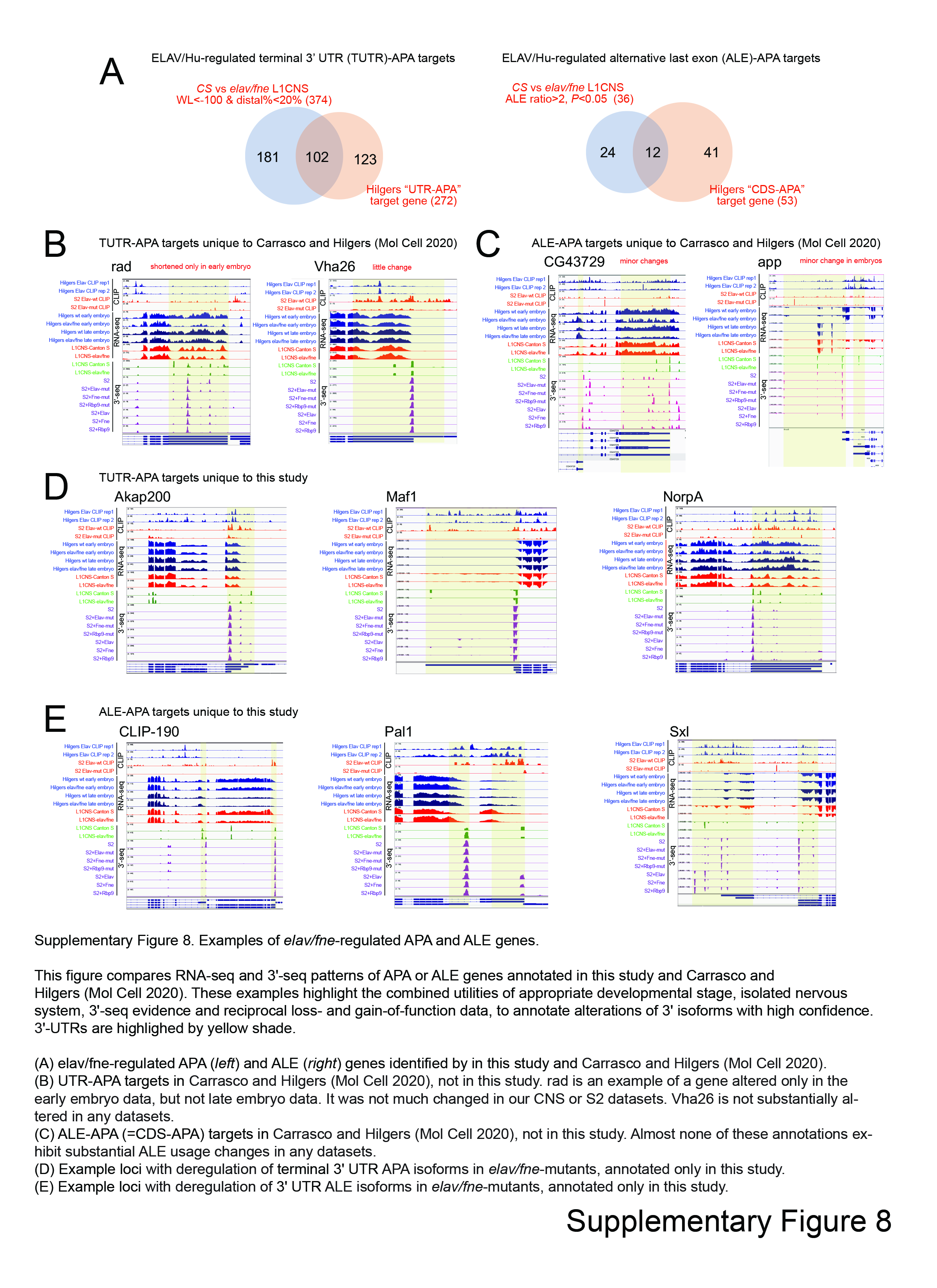

Supplement: S8 Fig — This Figure compares RNA-seq and 3’-seq patterns of APA or ALE genes annotated in this study and Carrasco and Hilgers (Mol Cell 2020). These examples highlight the combined utilities of appropriate developmental stage, isolated nervous system, 3’-seq evidence and reciprocal loss- and gain-of-function data, to annotate alterations of 3’ isoforms with high confidence. 3’-UTRs are highlighted by yellow shade. (A) elav/fne-regulated APA (left) and ALE (right) genes identified by in this study and Carrasco and Hilgers (Mol Cell 2020). (B) UTR-APA targets in Carrasco and Hilgers (Mol Cell 2020), not in this study. rad is an example of a gene altered only in the early embryo data, but not late embryo data. It was not much changed in our CNS or S2 datasets. Vha26 is not substantially altered in any datasets. (C) ALE-APA (= CDS-APA) targets in Carrasco and Hilgers (Mol Cell 2020), not in this study. Almost none of these annotations exhibit substantial ALE usage changes in any datasets. (D) Example loci with deregulation of terminal 3’ UTR APA isoforms in elav/fne-mutants, annotated only in this study. (E) Example loci with deregulation of 3’ UTR ALE isoforms in elav/fne-mutants, annotated only in this study. (TIF) [file pgen.1009439.s008.tif]

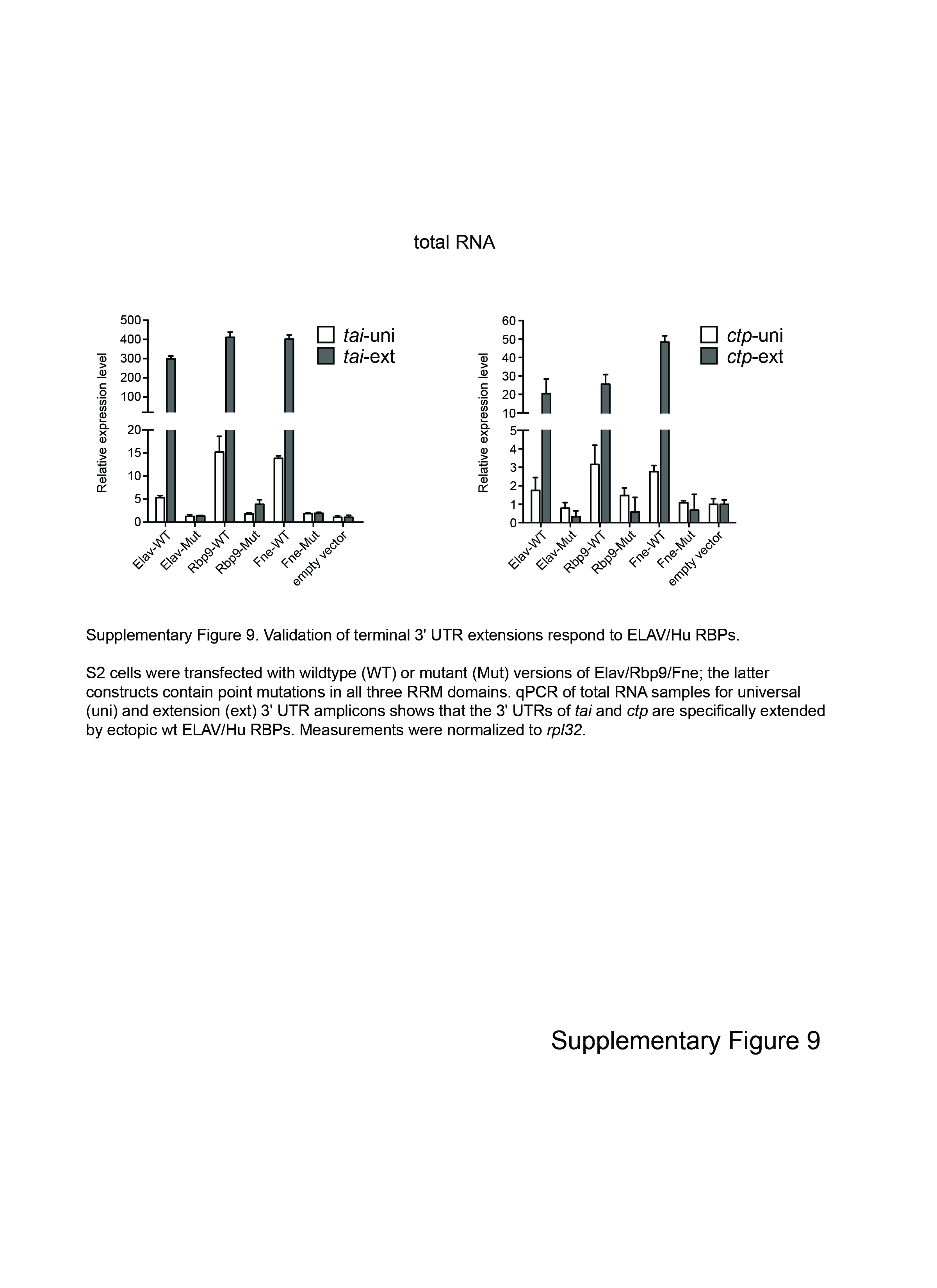

Supplement: S9 Fig — S2 cells were transfected with wildtype (WT) or mutant (Mut) versions of Elav/Rbp9/Fne; the latter constructs contain point mutations in all three RRM domains. qPCR of total RNA samples for universal (uni) and extension (ext) 3’ UTR amplicons shows that the 3’ UTRs of tai and ctp are specifically extended by ectopic wt ELAV/Hu RBPs. Measurements were normalized to rpl32. (TIF) [file pgen.1009439.s009.tif]

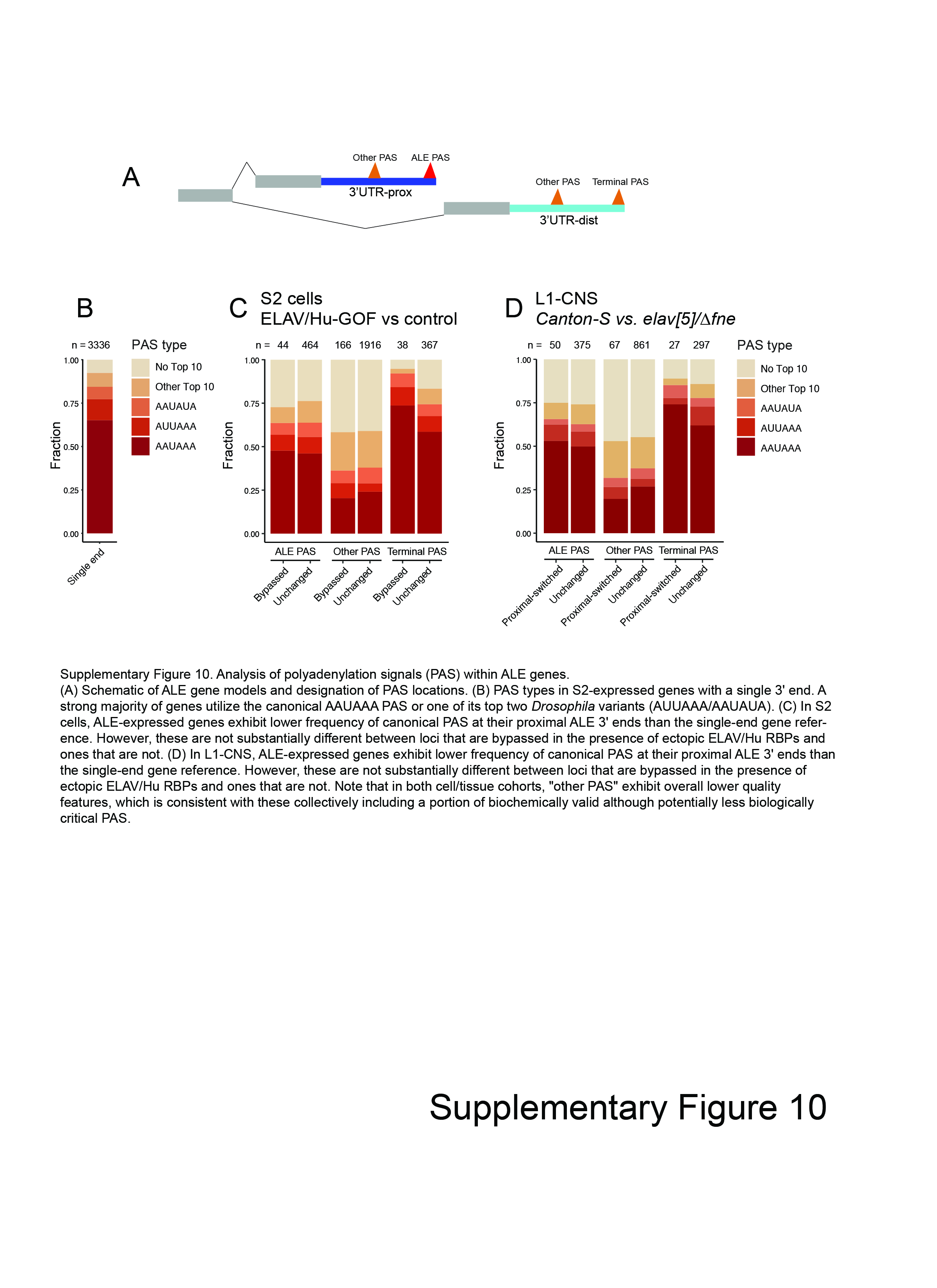

Supplement: S10 Fig — (A) Schematic of ALE gene models and designation of PAS locations. (B) PAS types in S2-expressed genes with a single 3’ end. A strong majority of genes utilize the canonical AAUAAA PAS or one of its top two Drosophila variants (AUUAAA/AAUAUA). (C) In S2 cells, ALE-expressed genes exhibit lower frequency of canonical PAS at their proximal ALE 3’ ends than the single-end gene reference. However, these are not substantially different between loci that are bypassed in the presence of ectopic ELAV/Hu RBPs and ones that are not. (D) In L1-CNS, ALE-expressed genes exhibit lower frequency of canonical PAS at their proximal ALE 3’ ends than the single-end gene reference. However, these are not substantially different between loci that are bypassed in the presence of ectopic ELAV/Hu RBPs and ones that are not. Note that in both cell/tissue cohorts, "other PAS" exhibit overall lower quality features, which is consistent with these collectively including a portion of biochemically valid although potentially less biologically critical PAS. (TIF) [file pgen.1009439.s010.tif]

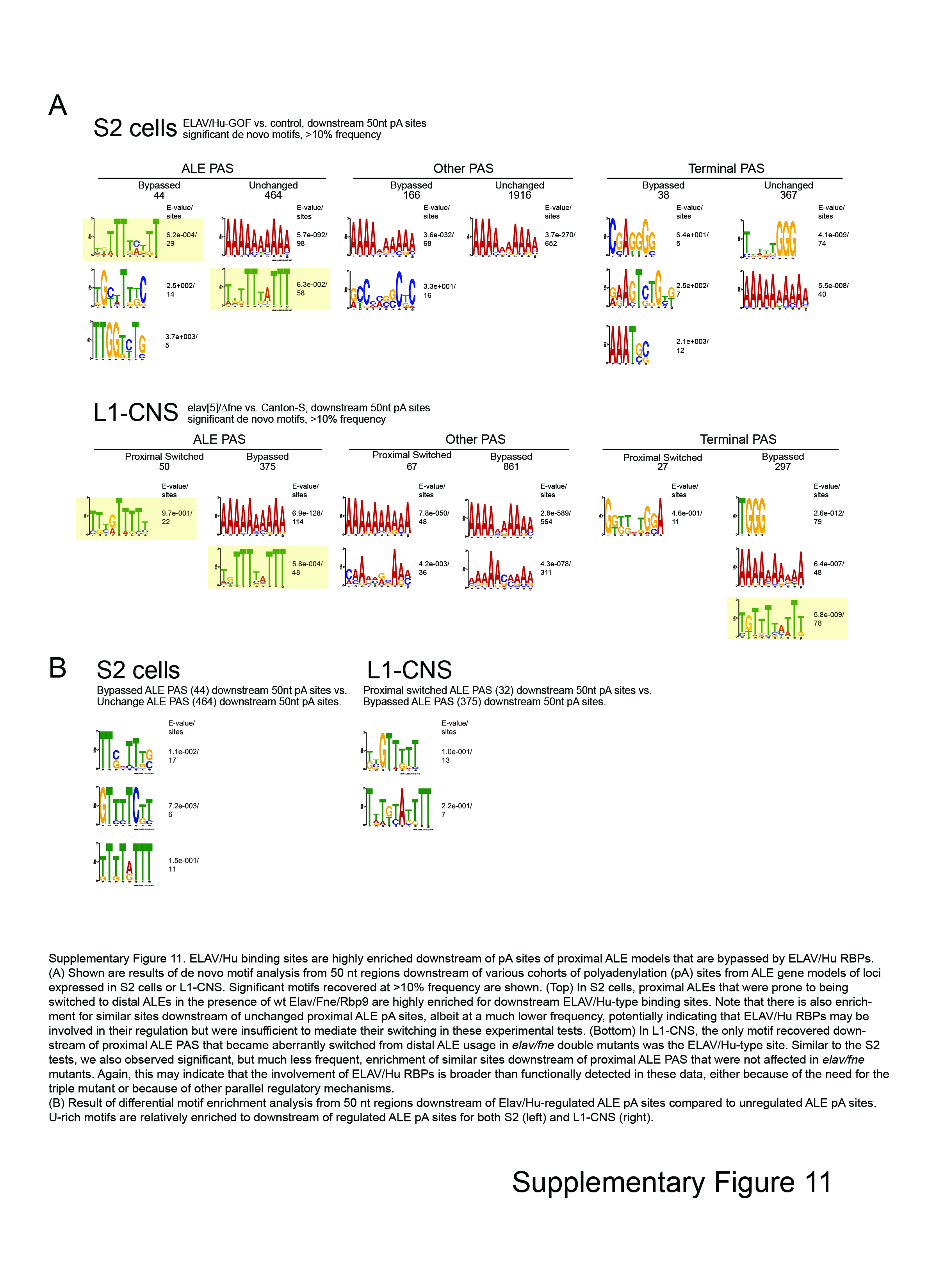

Supplement: S11 Fig — (A) Shown are results of de novo motif analysis from 50 nt regions downstream of various cohorts of polyadenylation (pA) sites from ALE gene models of loci expressed in S2 cells or L1-CNS. Significant motifs recovered at >10% frequency are shown. (Top) In S2 cells, proximal ALEs that were prone to being switched to distal ALEs in the presence of wt Elav/Fne/Rbp9 are highly enriched for downstream ELAV/Hu-type binding sites. Note that there is also enrichment for similar sites downstream of unchanged proximal ALE pA sites, albeit at a much lower frequency, potentially indicating that ELAV/Hu RBPs may be involved in their regulation but were insufficient to mediate their switching in these experimental tests. (Bottom) In L1-CNS, the only motif recovered downstream of proximal ALE PAS that became aberrantly switched from distal ALE usage in elav/fne double mutants was the ELAV/Hu-type site. Similar to the S2 tests, we also observed significant, but much less frequent, enrichment of similar sites downstream of proximal ALE PAS that were not affected in elav/fne mutants. Again, this may indicate that the involvement of ELAV/Hu RBPs is broader than functionally detected in these data, either because of the need for the triple mutant or because of other parallel regulatory mechanisms. (B) Result of differential motif enrichment analysis from 50 nt regions downstream of Elav/Hu-regulated ALE pA sites compared to unregulated ALE pA sites. U-rich motifs are relatively enriched to downstream of regulated ALE pA sites for both S2 (left) and L1-CNS (right). (TIF) [file pgen.1009439.s011.tif]

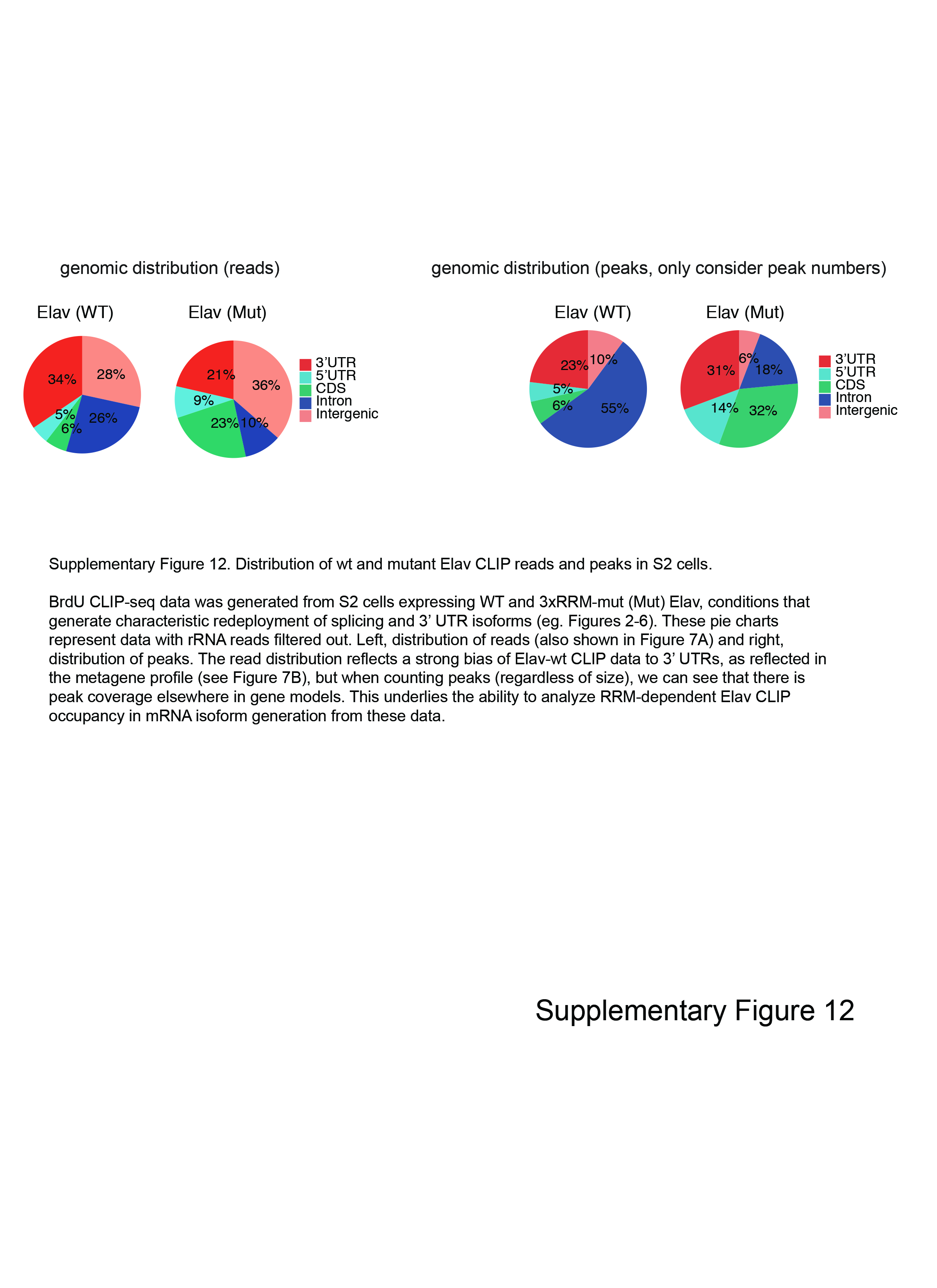

Supplement: S12 Fig — BrdU CLIP-seq data was generated from S2 cells expressing WT and 3xRRM-mut (Mut) Elav, conditions that generate characteristic redeployment of splicing and 3’ UTR isoforms (eg. Figs 2–6). These pie charts represent data with rRNA reads filtered out. Left, distribution of reads (also shown in Fig 7A) and right, distribution of peaks. The read distribution reflects a strong bias of Elav-wt CLIP data to 3’ UTRs, as reflected in the metagene profile (see Fig 7B), but when counting peaks (regardless of size), we can see that there is peak coverage elsewhere in gene models. This underlies the ability to analyze RRM-dependent Elav CLIP occupancy in mRNA isoform generation from these data. (TIF) [file pgen.1009439.s012.tif]

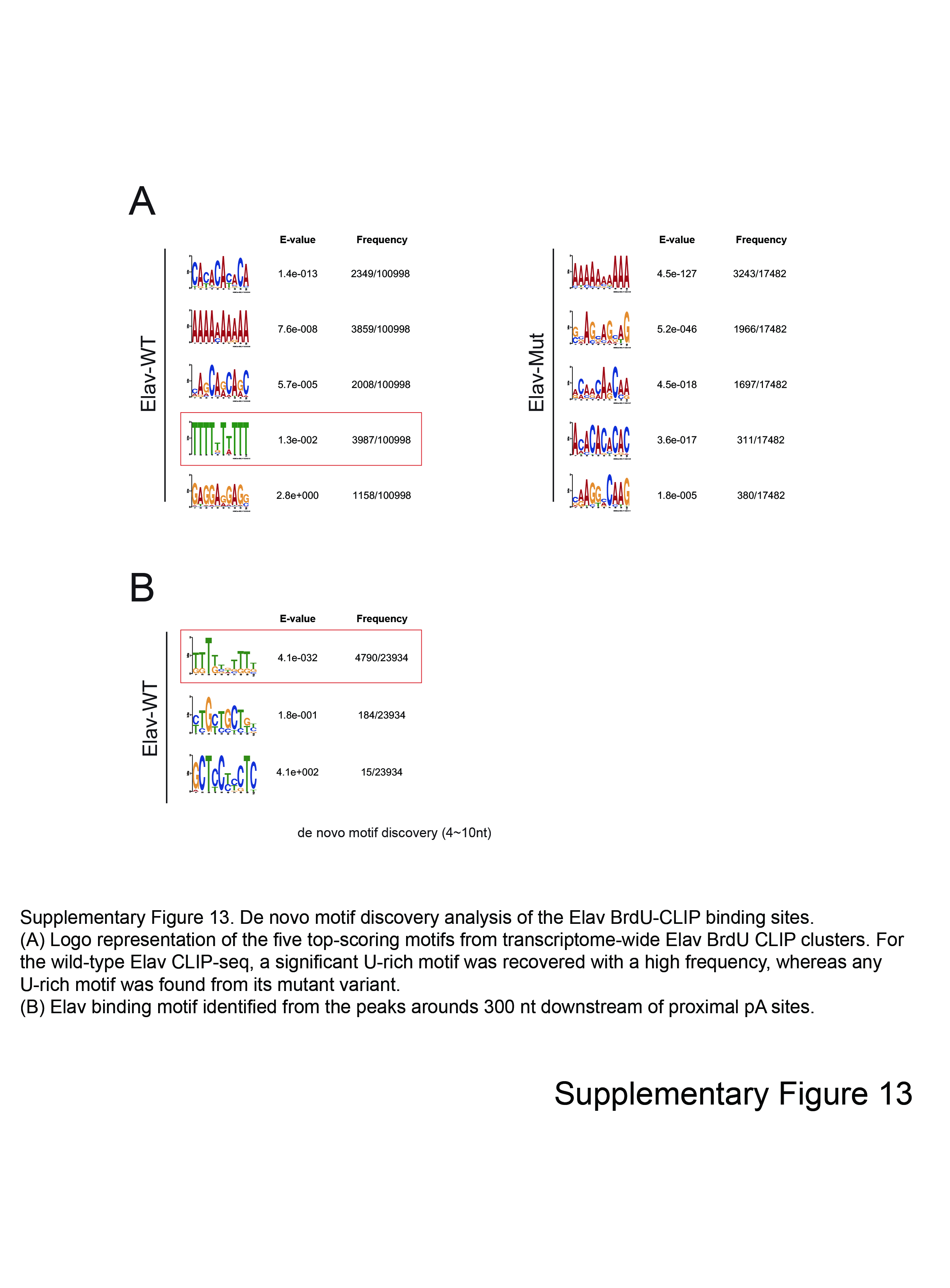

Supplement: S13 Fig — (A) Logo representation of the five top-scoring motifs from transcriptome-wide Elav BrdU CLIP clusters. For the wild-type Elav CLIP-seq, a significant U-rich motif was recovered with a high frequency, whereas any U-rich motif was found from its mutant variant. (B) Elav binding motif identified from the peaks arounds 300 nt downstream of proximal pA sites. (TIF) [file pgen.1009439.s013.tif]

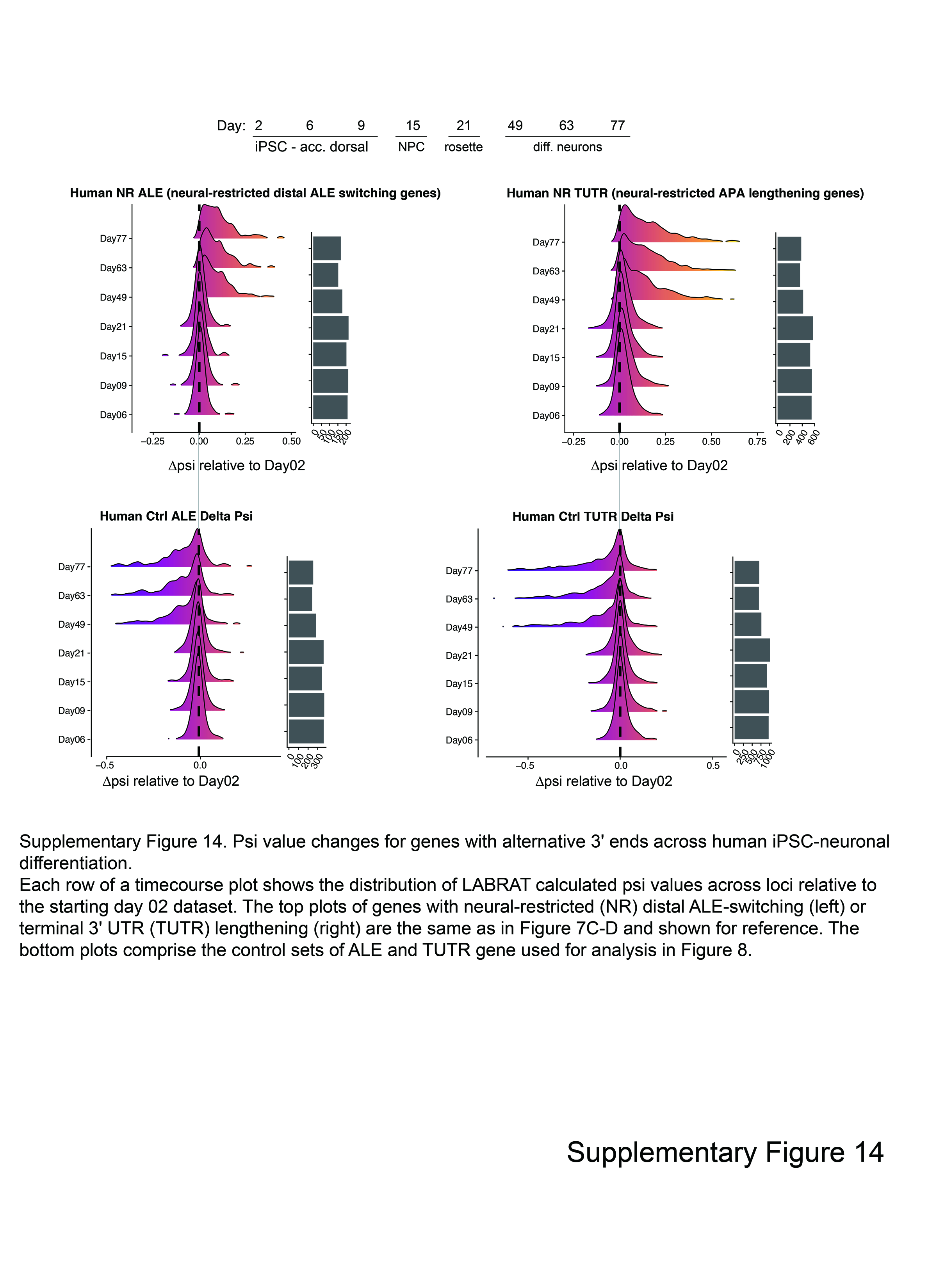

Supplement: S14 Fig — Each row of a timecourse plot shows the distribution of LABRAT calculated psi values across loci relative to the starting day 02 dataset. The top plots of genes with neural-restricted (NR) distal ALE-switching (left) or terminal 3’ UTR (TUTR) lengthening (right) are the same as in Fig 7C and 7D and shown for reference. The bottom plots comprise the control sets of ALE and TUTR gene used for analysis in Fig 8. (TIF) [file pgen.1009439.s014.tif]
